# *Drosophila* RpS12 controls translation, growth, and cell competition through Xrp1

**DOI:** 10.1371/journal.pgen.1008513

**Published:** 2019-12-16

**Authors:** Zhejun Ji, Marianthi Kiparaki, Virginia Folgado, Amit Kumar, Jorge Blanco, Gerard Rimesso, Jacky Chuen, Yang Liu, Deyou Zheng, Nicholas E. Baker

**Affiliations:** 1 Department of Genetics, Albert Einstein College of Medicine, Bronx, New York, United States of America; 2 The Saul R. Korey Department of Neurology, Albert Einstein College of Medicine, Bronx, New York, United States of America; 3 Dominick P. Purpura Department of Neuroscience, Albert Einstein College of Medicine, Bronx, New York, United States of America; 4 Department of Developmental and Molecular Biology, Albert Einstein College of Medicine, Bronx, New York, United States of America; 5 Department of Ophthalmology and Visual Sciences, Albert Einstein College of Medicine, Bronx, New York, United States of America; University of Colorado, UNITED STATES

## Abstract

Whereas complete loss of Rp function is generally lethal, most heterozygous *Rp* mutants grow more slowly and are subject to competitive loss from mosaics tissues that also contain wild type cells. The *rpS12* gene has a special role in the cell competition of other Ribosomal Protein (Rp) mutant cells in *Drosophila*. Elimination by cell competition is promoted by higher RpS12 levels and prevented by a specific *rpS12* mis-sense mutation, identifying RpS12 as a key effector of cell competition due to mutations in other *Rp* genes. Here we show that RpS12 is also required for other aspects of *Rp* mutant phenotypes, including hundreds of gene expression changes that occur in ‘Minute’ *Rp* heterozygous wing imaginal discs, overall translation rate, and the overall rate of organismal development, all through the bZip protein Xrp1 that is one of the RpS12-regulated genes. Our findings outline the regulatory response to mutations affecting essential Rp genes that controls overall translation, growth, and cell competition, and which may contribute to cancer and other diseases.

## Author summary

Ribosomes, the cellular machines that synthesizes new proteins, are themselves made of RNA and proteins. Mutations in Ribosomal protein (Rp) genes affect translation but they are also involved in human diseases and in cancer for reasons that are not yet clear. In the fruitfly *Drosophila melanogaster*, individual cells carrying mutations in *Rp* genes can be recognized and eliminated from tissues in a process of ‘cell competition’. Two genes that have been found to be required for cell competition include one specific Rp, RpS12 and the putative transcription factor Xrp1. Previous work has suggested that RpS12 was more specific for cell competition than Xrp1, which was also responsible for other aspects of *Rp* phenotypes such as changes in gene expression profiles, overall translation, and the rate of organismal development. This paper shows that RpS12 in fact controls Xrp1 levels and all the same aspects of the ‘Minute’ *Rp* heterozygous phenotype. Xrp1 is required for these RpS12 effects, establishing RpS12 as the most upstream component known of the signaling that occurs in *Rp* mutants.

## Introduction

Around 80 of the many proteins that interact with eukaryotic rRNA during ribosome biogenesis are stably associated with the mature ribosomal Large or Small Subunits [1]. Most of these ribosomal proteins (Rp) are essential to the cell, with roles in ribosome biogenesis and/or in the function of the mature ribosomes. A number of Rp also have additional functions, sometimes at extra-ribosomal locations [2, 3]. The full spectrum of Rp function is far from understood, as illustrated by the recurrent observation of Rp mutations in cancers. Frequent Rp point mutations occur in T-cell Acute Lymphoblastic Leukemia (T-ALL), Chronic Lymphocytic Leukemia (CLL), and colon cancer. Diamond Blackfan Anemia patients, at least 60% of whom carry germline mutations in Rp genes, exhibit higher rates of many cancers, as do patients with 5q syndrome, associated with somatically-acquired deficiency of the *RpS14* gene region [4–7]. It seems paradoxical that positive regulators of translation also behave like tumor suppressors.

To help understand the roles played by Rp, we have been studying Rp mutations in the fruitfly *Drosophila melanogaster*. As in other eukaryotes, homozygous mutation of most *Rp* loci is lethal in *Drosophila*. Heterozygotes for most *Rp* mutations have dominant phenotypes due to haploinsufficiency. In *Drosophila*, 66 of the 79 Rp are encoded by genes with a shared dominant ‘Minute’ phenotype, characterized by small adult macrochaetae (sensory bristles) and delayed development, and by other defects [8, 9]. These mutations also exhibit cell competition in mosaics, whereupon *Rp*^*+/-*^ cells can be eliminated in the presence of nearby wild type cells, which trigger their apoptosis [10–12]. In this paper we use the symbol ‘*Rp*’ to represent loci encoding ribosomal proteins that have the typical haploinsufficient phenotypes, but ‘*rp*’ for the small number of loci that are not haploinsufficient.

Genetic screens have identified mutations preventing cell competition, such as mutations in the putative transcription factor Xrp1 [13–15]. Whereas Xrp1 null mutations have no visible phenotype in otherwise wild type flies, Xrp1 expression is up-regulated cell-autonomously in *Rp*^*+/-*^ cells, and is required for their elimination ie clones of *Rp*^*+/-*^ cells are no longer eliminated by competition even in *Xrp1* heterozygotes [14, 16]. In the *Xrp1*^*-/-*^ background, even *Rp*^*-/-*^ cells survive and proliferate for some time [16]. Xrp1 is also known as a transcriptional target of p53 that is induced following irradiation, as part of a DNA-binding complex that binds to transposable P elements, and as a suppressor of a *Drosophila* model of Amyotrophic Lateral Sclerosis [17–20].

Xrp1 contributes to other aspects of the ‘Minute’ phenotype besides cell competition. Xrp1 is also responsible for the reduced growth rate of *Rp*^*+/-*^ cells, and for their reduced rate of translation, which remarkably therefore depends at least in part on a regulatory response to *Rp* mutations, and not on any direct effect of *Rp*^*+/-*^ genotypes on ribosome activity [16]. *Xrp1* is also responsible for some of the developmental delay typical of *Rp*^*+/-*^ genotypes, so that a more normal rate of development is restored when *Xrp1* is also mutated [16, 21]. Xrp1 mutations have only a small effect on the size of bristles, however [16]. *Rp*^*+/-*^ wing imaginal discs show a transcriptional signature distinct from the wild type [22], and more than 80% of these differences depend on Xrp1, corroborating the important role of Xrp1 in the Minute phenotype [16].

A second gene required for elimination of *Rp*^*+/-*^ cells by cell competition encodes the ribosomal protein RpS12. RpS12 is an essential ribosomal protein, whose null mutations are lethal as is typical for *Rp* genes, but is among the minority of *Drosophila* genes encoding Rp whose mutations are recessive, so that *rpS12*^+/-^ flies lack any visible ‘Minute’ phenotype [8, 23]. A homozygously viable mis-sense allele, *rpS12*^*D97*^, prevents cell competition of cells that carry mutations in other *Rp* genes [15, 23]. Multiple results indicate that *rpS12*^*D97*^ represents a loss-of-function mutation affecting a specific function of normal RpS12 in promoting cell competition that is genetically distinct from the essential role of RpS12 in cell growth and survival. For example, clones of cells over-expressing wild-type RpS12 in imaginal discs from ‘Minute’ *Rp*^*+/-*^ genotypes are eliminated during growth; by contrast, clones over-expressing the RpS12^D97^ protein survive normally, indicating that normal RpS12 protein has a function that is deleterious for *Rp*^*+/-*^ cells and that RpS12^D97^ lacks this function [23]. Similarly, cells carrying higher copy numbers of the *rpS12*^*+*^ gene are eliminated from ‘Minute’ genotypes heterozygous for mutations in other *Rp* genes, but cells carrying extra copies of the *rpS12*^*D97*^ allele are unaffected, again showing that *rpS12*^*D97*^ does not encode this aspect of wild type *rpS12*^*+*^ function. These and other studies indicate that in addition to an essential function, presumably in translation, the wild type RpS12 protein has another function in cell competition. The cell competition function is genetically separable because it is specifically affected by the Gly97Asp substitution [23].

Unlike Xrp1, the *rpS12*^*D97*^ mutation appeared not to rescue the developmental delay of *Rp* mutants (*RpL36*^*+/-*^ and *RpS18*^*+/-*^ were tested) [23]. Thus, the *rpS12*^*D97*^ mutation appeared more specific for the process of cell competition than mutations in *Xrp1* that affect multiple aspects of the Minute phenotype. This raised the possibility that whereas many genes that affect global aspects of the Minute phenotype would be affected in Xrp1 mutants, the subset whose expression depended on both Xrp1 and RpS12 might identify genes more specifically important in cell competition. It should be noted, however, that the elevated *Xrp1* expression in *Rp*^*+/-*^ cells was found to be RpS12-dependent, which is surprising if Xrp1 is upstream or parallel to RpS12 [24].

In order to resolve the relationship between Xrp1 and RpS12, and potentially to identify a subset of the Minute cell transcriptional signature that correlated specifically with cell competition rather than other aspects of the Minute phenotype, we compared the transcriptomes of wing discs from wild type *Xrp1*, and *rpS12*^*D97*^ genotypes and their combinations with *Rp*^*+/-*^ genotypes. These studies demonstrated clearly that *rpS12* acts upstream of *Xrp1* to control the *Rp*^*+/-*^ gene expression signature. Consistent with this, we also found that *rpS12* is required for the Xrp1-dependent reduction in overall translation of *Rp*^*+/-*^cells, and show through genetic epistasis that *rpS12* acts upstream of *Xrp1* in the regulation of imaginal disc cell translation and growth. We present new evidence that, although not previously appreciated, *rpS12* can affect the developmental delay of *Rp*^*+/-*^genotypes. The reason that the *rpS12*^*D97*^ mutation does not rescue *Rp*^*+/-*^developmental delay as dramatically as *Xrp1* mutations may be that *rpS12*^*D97*^ simultaneously retards development through a mechanism independent of Xrp1. Consistent with the notion that *rpS12*^*D97*^ has additional effects, we show that *rpS12*^*D97*^ diminishes longevity and can affect adult organ size.

Our findings provide clear molecular and genetic evidence that RpS12 plays a central and early role in generating multiple aspects of the *Rp*^*+/-*^ phenotype in *Drosophila* imaginal discs by activating Xrp1 expression and activity.

## Results

### RpS12 regulates the Xrp1-dependent gene expression program of *Rp*^*+/-*^mutant cells

**Fig 1:**
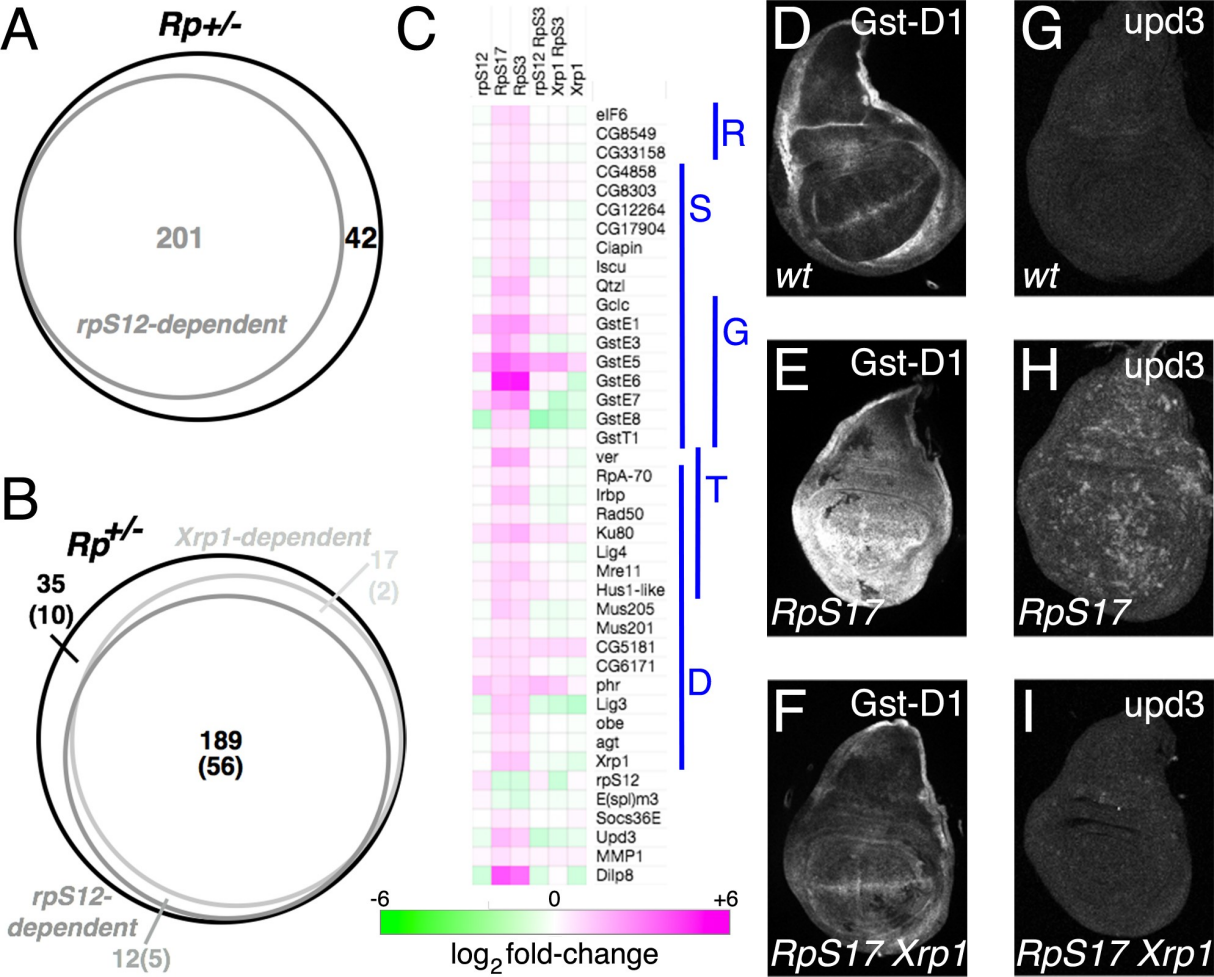
RpS12-dependent gene expression in *Rp*^*+/-*^ wing discs. A) 201 of 253 mRNAs altered in *Rp*^*+/-*^ wing discs were *rpS12*-dependent. B) *rpS12*- and *Xrp1-*dependent *Rp*^*+/-*^ wing disc genes were largely overlapping. Shown in parentheses are the numbers of genes with predicted Xrp1 binding motifs (See also S3 Table). C. Heat map of fold changes in mRNA levels between wing discs from wild type and from indicated genotypes. Upregulation in *Rp*^*+/-*^ genotypes was overwhelmingly dependent on both *rpS12* and *Xrp1*. Genes shown here include all those corresponding to the enriched GO terms mature ribosome assembly (R), sulfur compound metabolic process (S), glutathione metabolic process (G), telomere maintenance (T), and DNA repair (D). The individual fold changes are shown in the S1 Table and S2 Table. Panels D-F show GstD1-LacZ expression in wing discs. Panels G-I show Upd3.3-LacZ labeling of wing discs. Genotypes: wild type (D,G); *M(3)i*^*55*^ *ubi-GFP FRT80B*/+ (E,H); *M(3)i*^*55*^ *ubi-GFP FRT80B*/*FRT82 Xrp1*^*m2-73*^ (F,I).

*Rp*^*+/-*^ wing discs exhibit an altered pattern of transcription that is thought to encode some of their properties [22]. Our studies identified 253 mRNA’s that were altered in the same direction in both *RpS3*^*+/-*^ and *RpS17*^*+/-*^ genotype [24]. To identify the RpS12-dependent changes, we now report mRNA-Seq analysis from *rpS12*^*D97/D97*^ *RpS3*^*+/-*^ wing imaginal discs, as well as *rpS12*^*D97/D97*^ and *Xrp1*^*+/-*^ wing imaginal discs. Strikingly, 201 of the 253 genes (79%) changing expression in Minute *Rp*^*+/-*^ genotypes did so in response to *rpS12* activity (Fig 1A). *Xrp1* also regulates most of the differential mRNA expression in Minute *Rp*^*+/-*^ wing discs [24]. Of the 201 RpS12-regulated genes, differential expression of 189 (94%) also depended on Xrp1, strongly suggesting that RpS12 and Xrp1 affected a common pathway (Fig 1B).

We found previously that *Rp*^*+/-*^-dependent transcripts were enriched for GO terms glutathione metabolic process, telomere maintenance, DNA recombination, and iron-sulfur cluster assembly [24]. A more recent GO term database also revealed enrichment for ‘mature ribosome assembly’, reflecting elevated transcript levels for the *eIF6*, *CG8549* and *CG33158* genes. Our RpS12- and Xrp1-dependent transcript datasets were both enriched for the same GO terms. The genes with the enriched GO terms were overwhelmingly co-regulated by RpS12 and Xrp1 (Fig 1C; S1 Table).

Most apparent differences between our results and gene expression changes in *RpS3*^*+/-*^ wing imaginal discs reported by another group [22] only reflect different statistical cutoffs (see methods). By our criteria, the differentially-expressed genes reported previously [22] were significantly enriched for the GO terms oxidation-reduction process, glutathione metabolic process, telomere maintenance, and sensory perception of sweet taste, very similar to our own results.

Despite hypotheses that *Rp*^*+/-*^ cells might be less efficient at capturing Dpp [25], or experience elevated Wg signaling [26], representative target genes for Dpp and Wg signals were not affected in *RpS3*^*+/-*^ and *RpS17*^*+/-*^ wing discs. There were also no gene expression changes of signature genes for Ras, Hh, or Salvador-Warts-Hippo signaling (S2 Table). *Socs36E*, a target of Jak/Stat signaling was significantly elevated, however, as was a target of JNK signaling (*MMP1*) and one Notch target gene (*E(spl)-m3*) was decreased, although other Notch targets were unaffected (Fig 1C, S2 Table). We confirmed that *Rp*^+/-^ cells exhibit an oxidative stress response using the *GstD1-LacZ* reporter line, whose expression was elevated in *RpS17*^*+/-*^ wing discs in an *Xrp1*-dependent manner (Fig 1D–1F), and that a Upd-3 reporter also elevated in an Xrp1-dependent manner, which would be expected to signal through Jak/Stat (Fig 1G–1I). We had reported previously that expression of the JNK reporter *puc-LacZ* in *RpS18*^*+/-*^ cells was dependent on *Xrp1* [24]. We also found that the loci whose expression was affected in Minute Rp^+/-^ genotypes were enriched for Xrp1 binding motifs. Xrp1 binding sites were among the most highly enriched binding sites in these genes, identified near 77 of these 253 genes (30%)(Fig 1B; S3 Table). ChIP experiments would be required to confirm that these genes were direct Xrp1 targets, however.

We examined transcript levels of the rpS12 and Xrp1 genes themselves to see how they might be related. *rpS12* transcript levels were increased in *rpS12*^*D97/D97*^ wing imaginal discs compared to wild type, and reduced in both Minute *Rp*^*+/-*^ genotypes (Fig 1C, S1 Table). Because *rpS12* transcript levels were elevated in *rpS12*^*D97/D97*^ *RpS3*^*+/-*^ wing imaginal discs, we conclude that reduction in *RpS3*^*+/-*^ wing discs depended on normal RpS12 function (Fig 1C, S1 Table). Because the *rpS12*^*D97*^ allele affects the coding sequence, this suggests that RpS12 protein might regulate expression of its own mRNA, either directly or indirectly, which could also explain elevated *rpS12* mRNA levels in the *rpS12*^*D97/D97*^ genotype. By contrast, *rpS12* mRNA levels were not different between *RpS3*^*+/-*^ wing discs and *RpS3*^*+/-*^ *Xrp1*^*+/-*^ wing discs, so Xrp1 was not responsible for altered *rpS12* expression in this Minute Rp^+/-^ genotype (Fig 1C, S1 Table).

As reported previously, *Xrp1* transcription is elevated in Minute *Rp*^*+/-*^ genotypes (Fig 1C, S1 Table) [14, 22, 24]. This increase in *Xrp1* transcripts was entirely dependent on *rpS12* function (Fig 1C, S1 Table), which is consistent with the reported RpS12-dependence of Xrp1 protein and Xrp1-LacZ enhancer trap expression in *RpS17*^*+/-*^ or *RpS18*^*+/-*^ wing discs [24]. Xrp1 mRNA levels also depended on *Xrp1* function, implying an autoregulatory loop of *Xrp1* expression in Rp^+/-^ cells (Fig 1C, S1 Table).

Taken together, these results strongly suggest that RpS12 is an upstream regulator of the *Rp*^*+/-*^ phenotype. They indicate that RpS12 activity elevates Xrp1 expression in Minute Rp^+/-^ genotypes, which in turn is directly or indirectly responsible for most of the transcriptional changes seen in Minute Rp^+/-^ wing discs.

### RpS12 affects translation and growth through Xrp1

**Fig 2:**
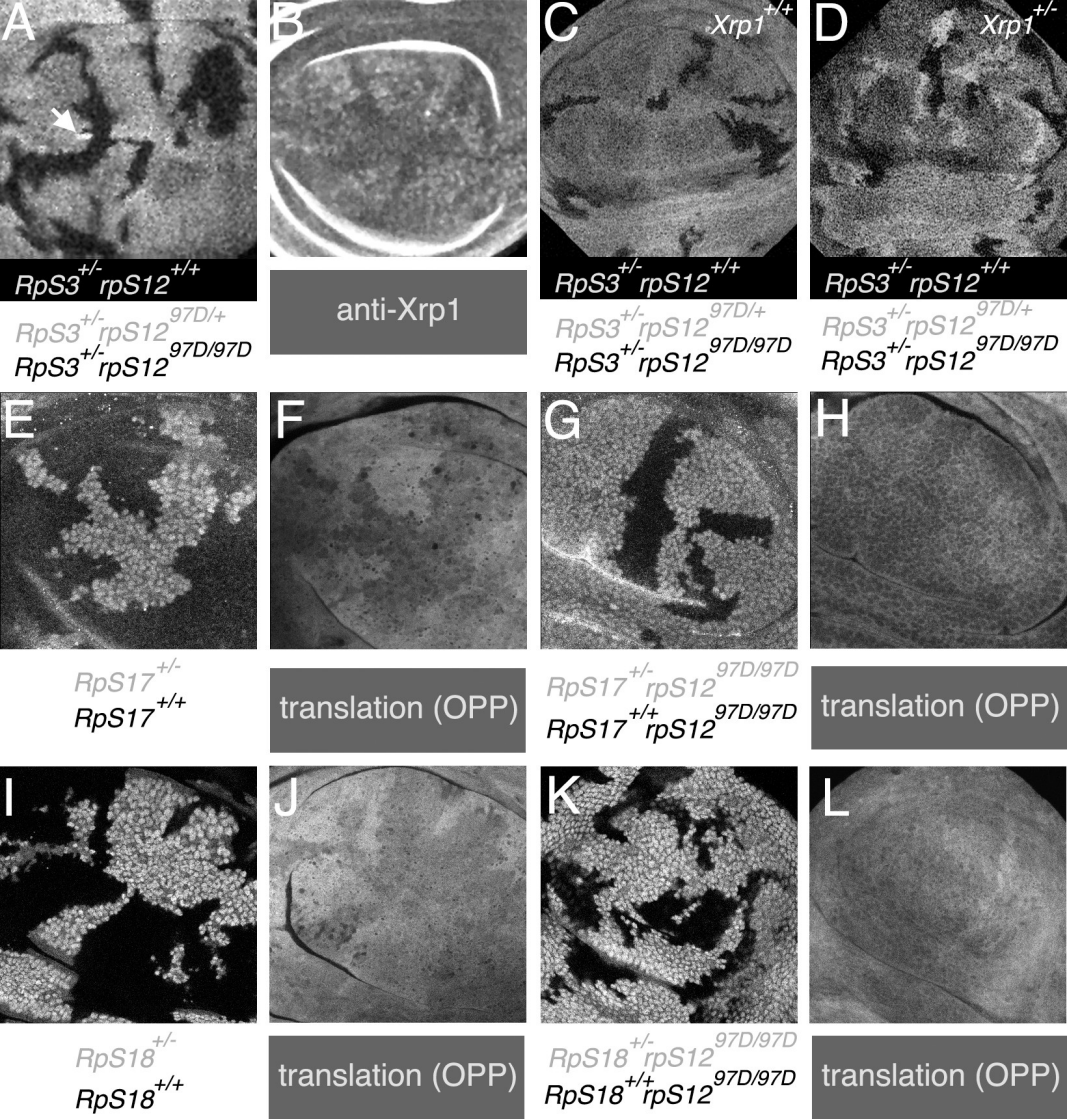
Growth and translation in *Rp*^*+/-*^ wing discs. All panels show wing pouch regions of third-instar wing imaginal discs. Genotypes are as indicated below each figure; the font colors correspond to the labeling of the corresponding genotype (the genotype corresponding to the most brightly labeled cells is shown in white on a black background). A,B). Xrp1 protein levels were lower in *RpS3*^*+/-*^ *rpS12*^*D97/D97*^ clones than in *RpS3*^*+/-*^ *rpS12*^*D97/+*^ cells. Small *RpS3*^*+/-*^ *rpS12*^*+/+*^ clones were detected rarely (eg arrow) C) *RpS3*^*+/-*^ *rpS12*^*+/+*^ clones are rarely detected in *RpS3*^*+/-*^ *rpS12*^*D97/+*^ wing discs, unlike the reciprocal *RpS3*^*+/-*^ *rpS12*^*D97/D97*^ clones. D) In contrast to panel C, in the *Xrp1*^*+/-*^ background *RpS3*^*+/-*^ *rpS12*^*+/+*^ clones were recovered similarly to the reciprocal *RpS3*^*+/-*^ *rpS12*^*D97/D97*^ clones. E,F). Overall translation was reduced in *RpS17*^*+/-*^ cells, as was described previously [16]. G,H). Overall translation was not affected in *RpS17*^*+/-*^ cells in *rpS12*^*D97/D97*^ wing discs. I,J) Translation in RpS18+/- cells is reduced compared to wt cells, as was described previously [16]. K,L). Further data related to this figure is shown in the S1 Fig. Genotypes: A-C) y w hsF; rpS12^D97^ FRT80B/P{arm-LacZ} FRT80B M(3)95A armLacZ. D) y w hsF; rpS12^D97^ FRT80B/P{arm-LacZ} FRT80B Xrp1^m2-73^ M(3)95A. E,F) y w hsF; RpS17^4^ P{ubi-GFP} FRT80B/FRT80B. G,H) y w hsF; rpS12^D97^ RpS17^4^ P{ubi-GFP} FRT80B/rpS12^D97^ FRT80B. I,J) y w hsF; FRT42D P{arm-LacZ} M(2)56i/FRT42D. K,L) y w hsF; FRT42D P{arm-LacZ} M(2)56i/FRT42D; rpS12^D97^ FRT80B/rpS12^D97^ FRT80B.

Genetic mosaic experiments were performed to determine whether RpS12 regulates *Xrp1* expression cell-autonomously. Clones of *RpS3*^*+/-*^ *rpS12*^*D97/D97*^ cells were induced in *RpS3*^*+/-*^ *rpS12*^*D97/+*^ wing discs. The *RpS3*^*+/-*^ *rpS12*^*D97/D97*^ cells showed lower levels of Xrp1 protein expression, showing that RpS12 regulated Xrp1 levels cell-autonomously (Fig 2A and 2B).

To confirm that Xrp1 functions downstream of RpS12, as suggested by the expression data, we tested whether Xrp1 was required for RpS12 to influence cell competition. In the experiment described above and shown in Fig 2A and 2B, the *RpS3*^*+/-*^ cells that have two copies of the wild type *rpS12* allele (*RpS3*^*+/-*^ *rpS12*^*+/+*^) are frequently lost in the *RpS3*^*+/-*^ *rpS12*^*D97/+*^ background because their higher copy number of the *rpS12*^*+*^ locus targets them for elimination by cell competition (Fig 2A and 2C) [23]. This elimination depended on Xrp1, because when *Xrp1* was mutated the *RpS3*^*+/-*^ *rpS12*^*+/+*^ *Xrp1*^*+/-*^ cells survived in the *RpS3*^*+/-*^ *rpS12*^*D97/+*^ *Xrp1*^*+/-*^ wing discs (Fig 2D). Therefore, RpS12 needs Xrp1 to affect *RpS3*^*+/-*^ clone survival.

If RpS12 is the upstream regulator of Xrp1 expression, it should be required for the whole Xrp1-dependent response. Xrp1 is required for the slow growth of Minute Rp^+/-^ genotypes at the cellular level, and regulates their global translation rate. Minute Rp^+/-^ genotypes exhibit slower overall translation rate than wild type cells, and this depends on Xrp1 because normal translation is largely restored in *Rp*^*+/-*^ *Xrp1*^*+/-*^ cells [24]. If RpS12 acts upstream of Xrp1, we would expect that in *rpS12*^*D97*^ mutants there would also be no difference in translation rate between wild type and *Rp*^*+/-*^ cells. As predicted, whereas *RpS17*^*+/-*^ and *RpS18*^*+/-*^ cells exhibited reduced translation rate compared to wild type cells in the presence of wild type *rpS12*, both *RpS17*^*+/-*^ and *RpS18*^*+/-*^ cells showed translation indistinguishable from *RpS17*^*+/+*^ and *RpS18*^*+/+*^ cells in the *rpS12*^*D97/D97*^ genotype(Fig 2E–2L; S1 Fig).

To address this question in another way, *rpS12*^*D97/D97*^ *RpS3*^*+/-*^ and *rpS12*^*+/+*^ *RpS3*^*+/-*^ clones were examined in *rpS12*^*D97/+*^ *RpS3*^*+/-*^ wing discs. As noted above, *rpS12*^*+/+*^ *RpS3*^*+/-*^ clones were infrequent and always small, and they generally exhibited reduced translation (S2 Fig panels A,B). The *rpS12*^*D97/D97*^ *RpS3*^*+/-*^ cells had higher rates of translation that could often be distinguished from the *rpS12*^*D97/+*^ *RpS3*^*+/-*^ background (S2 Fig panels A,B). These *rpS12*-dependent differences were abolished in the *Xrp1*^*+/-*^ background (S2 Fig panel C). We also found that translation in *RpS17*^*+/-*^ *rpS12*^*D97/+*^ was quite similar to *RpS17*^*+/+*^(S2 Fig panels D-F).

Taken together, these experiments showed that *rpS12* requires *Xrp1* to affect the competitiveness of *Rp*^*+/-*^cells, and that the reduction in translation that is mediated by Xrp1 is also downstream of RpS12. These findings strongly support the conclusion derived from gene expression studies: RpS12 appears to be the upstream regulator that signals to Xrp1 in *Rp*^*+/-*^genotypes, and accordingly it is required for Xrp1-dependent effects on translation and growth as well as for cell competition.

### RpS12 also makes Xrp1-independent contributions to the rate of development

If RpS12 is the upstream regulator of Xrp1, it is surprising that *rpS12* would not affect *Rp*^*+/-*^ developmental delay, since Xrp1 is responsible for much of this delay [23, 24]. RpS12 regulates Xrp1 expression, and through Xrp1 gene expression, translation and cell competition in Rp^+/-^ genotypes. Therefore, *rpS12* mutations should affect the Rp^+/-^ developmental delay like *Xrp1* mutations do.

**Fig 3:**
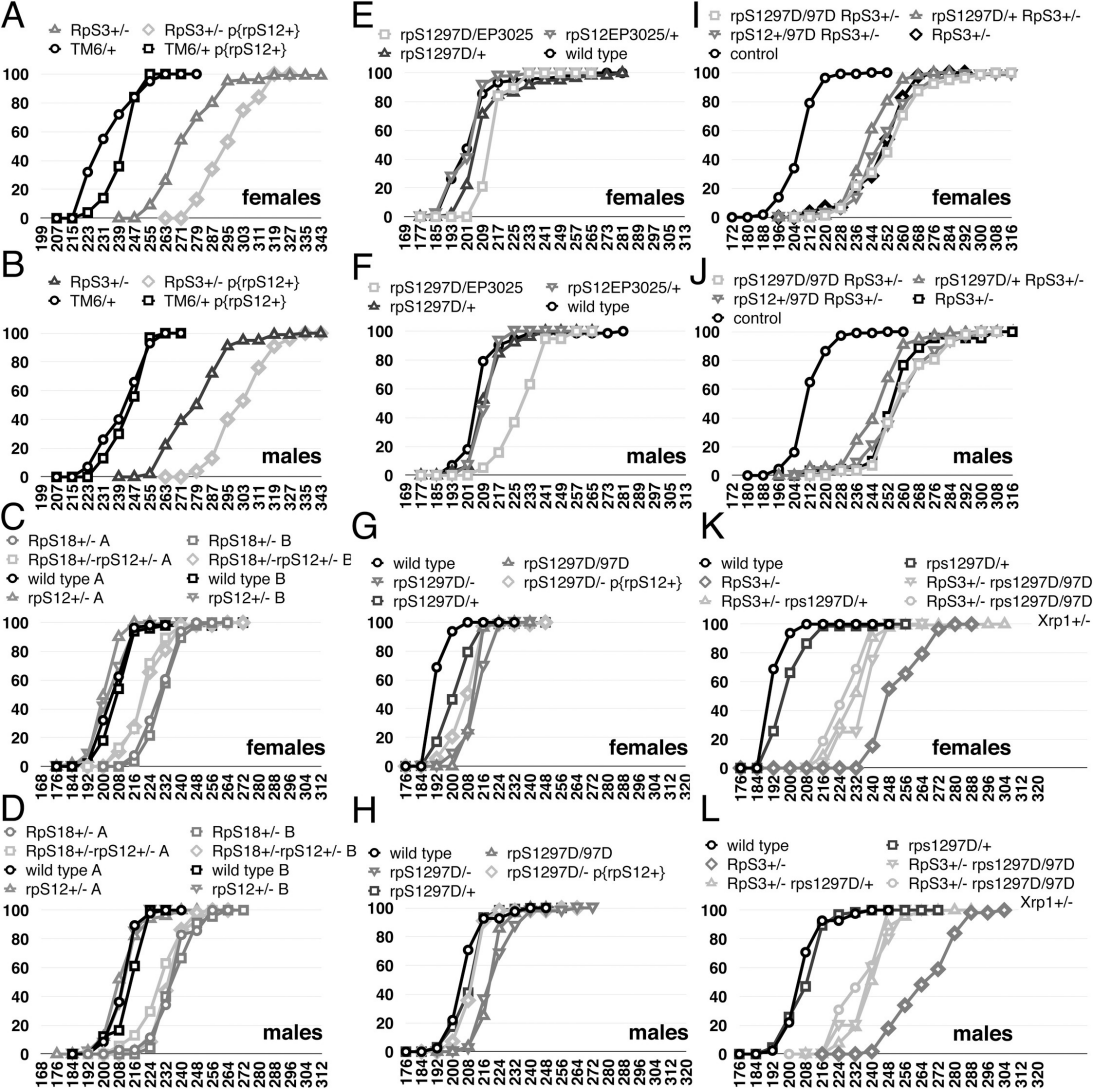
Contribution of RpS12 to rate of development. All panels show the cumulative percentage of adult flies emerged according to time after egg laying in hours. A,B) A genomic transgene including the *rpS12*^*+*^ locus had little effect on wild type development but significantly retarded development of *RpS3*^*+/-*^ flies. C,D) Heterozygosity for *rpS12* had little effect on wild type development but modestly accelerated development of *RpS18*^*+/-*^ flies. E,F) the genotype *rpS12*^*D97/EP3025*^ modestly delayed development of otherwise wild type flies. G,H) the genotype *rpS12*^*D97/D97*^ modestly delayed development of otherwise wild type flies and this delay was suppressed by a genomic transgene including the *rpS12*^*+*^ locus. I,J) An experiment in which *rpS12*^*D97/D97*^ and *rpS12*^*D97/+*^ genotypes had little effect on the development of *RpS3*^*+/-*^ flies. K,L) An experiment in which *rpS12*^*D97/D97*^ and *rpS12*^*D97/+*^ genotypes suppressed the developmental delay of *RpS3*^*+/-*^ flies, to a similar extent to *RpS3*^*+/-*^ *rpS12*^*D97/D97*^ *Xrp1*^*+/-*^ flies. In Fig 3K and 3L, the development of *RpS3*^*+/-*^ *rpS12*^*D97/D97*^ flies and *RpS3*^*+/-*^ *rpS12*^*D97/D97*^ *Xrp1*^*+/-*^ flies are significantly faster than *RpS3*^*+/-*^ flies (p<0.00001 in all cases, Mann Whitney procedure). The detailed genotypes used and numerical data corresponding to these graphs is tabulated in the S4 Table.

To revisit the effects of RpS12 on developmental rate, we looked at the effects of increased *rpS12* gene dose on Minute Rp^+/-^ genotypes. Compared to wild type controls, *RpS3*^*+/-*^ females emerged 24h later, on average, and *RpS3*^*+/-*^ males 28h later, on average (Fig 3A and 3B). An additional copy of the *rpS12*^*+*^ locus, encoded on a transgene, further delayed adult emergence by another 24h in *rpS12*^*+/+/+*^ *RpS3*^*+/-*^ females, on average, and by another 20h in males (Fig 3A and 3B). No *RpS3*^*+/-*^ adults carrying two extra copies of the *rpS12*^*+*^ locus were recovered, although the *rpS12*^*+*^ transgene is homozygously viable by itself. Therefore, although they had no effects in Rp^+/+^ genotypes, extra copies of *rpS12*^*+*^ exacerbated the *Rp*^*+/-*^ developmental delay, showing that RpS12 can indeed regulate developmental rate.

We then re-examined the effect of *rpS12* loss of function in developmental delay. Previously, we reported that *RpS18*^*+/-*^ *rpS12*^*D97/-*^ flies develop at the same rate as *RpS18*^*+/-*^ *rpS12*^*D97/+*^ flies and *RpS18*^*+/-*^ *rpS12*^*+/+*^ flies [23]. We noticed, however, that in the same experiments there was a small suppression of developmental delay of *RpS18*^*+/-*^ flies that were also *rpS12*^*+/-*^ (Fig 3C and 3D). In addition, we previously observed a small developmental delay of *rpS12*^*D97/-*^ flies in comparison to wild type [23]. Because this effect was at the limit of what was observable (only 4-5h), and its significance might be questioned, we also examined other *rpS12* genotypes. We confirmed a small developmental delay in *rpS12*^*D97/EP3025*^, a different *rpS12*^*D97/-*^ genotype from the *rpS12*^*D97/s2783*^ used previously, and in *rpS12*^*D97/D97*^ (Fig 3E–3H). Finally, we found that the developmental delay of *rpS12*^*D97/D97*^ flies was at least partially suppressed by an *rpS12*^*+*^ genomic transgene, suggesting that the mutant *rpS12* genotype was at least partly responsible (Fig 3G and 3H). Taken together, these results suggest that the *rpS12*^*D97*^ mutation delays development of otherwise wild type flies, although to a milder degree than the dominant effect of ‘Minute’ mutations at other *Rp* loci.

**Fig 4:**
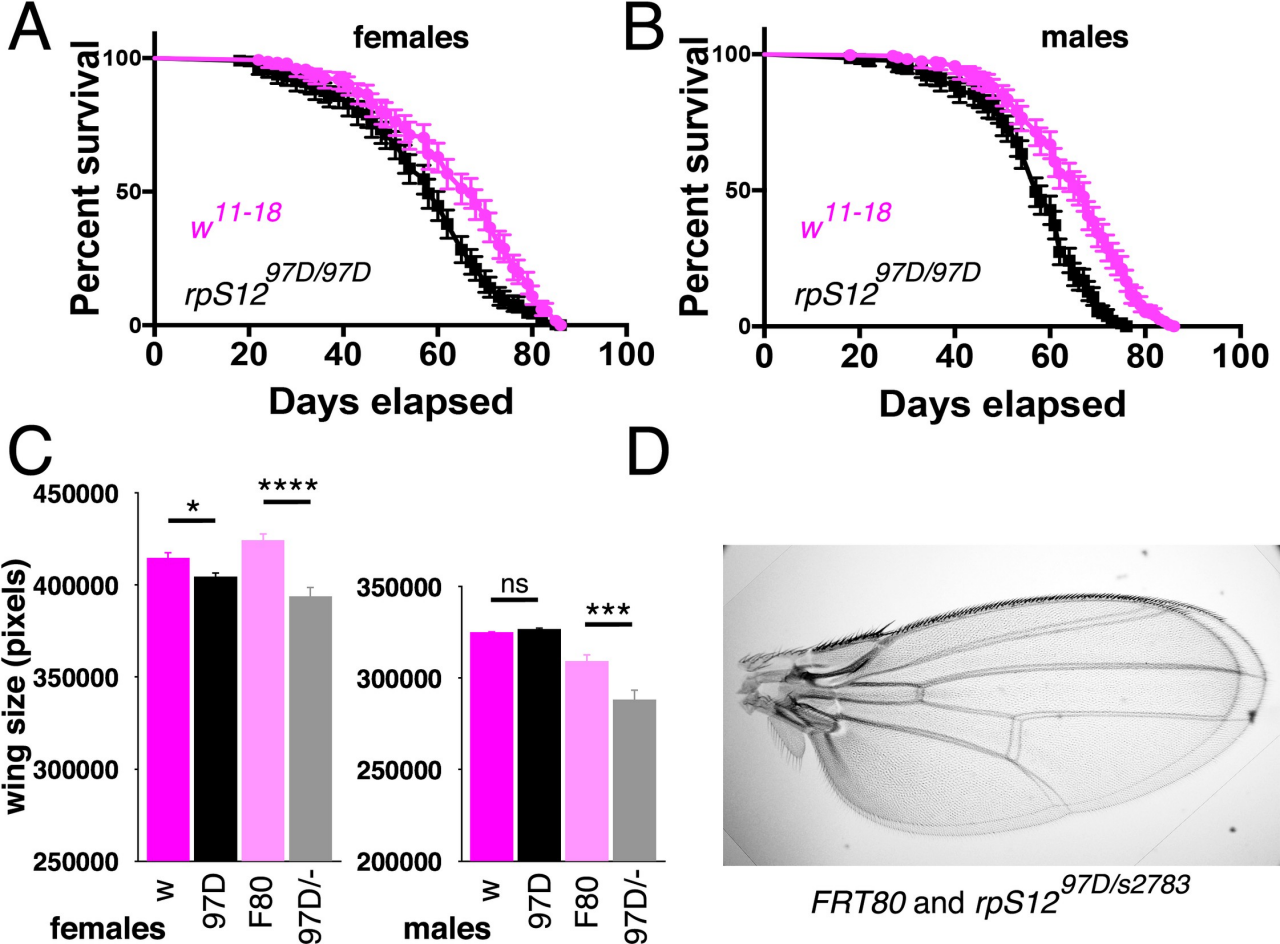
Contributions of RpS12 to longevity and organ size. A) Survival curve of female *rpS12*^*G97D*^ flies compared to *w*^*11-18*^ (wild type) controls. Shown is the mean are 95% confidence limits from 3 replicates of 120 flies each. B) Survival curve of male *rpS12*^*G97D*^ flies compared to *w*^*11-18*^ (wild type) controls. The difference is significant at p<0.0001 Log-rank (Mantel-Cox) test. See the S3 Fig for individual replicates and the S5 Table for raw data. C) Adult wing size was significantly smaller for the *rpS12*^*G97D*^ females compared to *w*^*11-18*^ (p = 0.0165, two-tailed Welch’s t-test) and for *rpS12*^*G97D/-*^ females compared to FRT80B (p = 0.000006, two-tailed Welch’s t-test). Wing size was also smaller for *rpS12*^*G97D/-*^ males compared to FRT80B (p = 0.00014, two-tailed t-test) but not for *rpS12*^*G97D*^ males compared to *w*^*11-18*^ (p = 0.126, two-tailed t-test). Raw data are tabulated in the S7 Table. D) Wings from *rpS12*^*G97D/-*^ and FRT80B females overlaid to illustrate the difference. The *rpS12*^*G97D/-*^ wing is smaller.

Further evidence for a detrimental *rpS12*^*D97*^ phenotype came from studies of longevity and organ size. To compare the longevity of *rpS12*^*D97*^ flies with a control in the same genetic background, we backcrossed the *rpS12*^*D97*^ mutation into a *w*^*11-18*^ background for 6 generations, then established homozygous viable *rpS12*^*D97*^ and wild type lines from single sibling flies from the last generation. In this background the median lifespan of *rpS12*^*D97*^ mutant females was 65 days, compared to 72 days in control females (Fig 4A; S3 Fig). The median lifespan of *rpS12*^*D97*^ mutant males was 58 days, compared to 67 days in controls (Fig 4B). The median lifespan of *rpS12*^*D97*^ mutant These differences were statistically significant. We also noticed that *rpS12*^*D97*^ homozygous adults and adults from a *rpS12*^*D97/-*^ genotype often had smaller wings, reduced by up to 8% of total area (Fig 4C and 4D).

If the *rpS12*^*D97*^ mutation affects non-Minute flies, this suggests how *rpS12*^*D97*^ *Rp*^*+/-*^ flies might develop more slowly than *Xrp1*^*+/-*^ *Rp*^*+/-*^ flies. The *rpS12*^*D97*^ mutation may both suppress *Rp*^*+/-*^ developmental delay by preventing Xrp1 expression, at the same time as delaying development independently by another mechanism. Results obtained with *RpS3*^*+/-*^ could be consistent with this hypothesis. In multiple experiments, *rpS12*^*D97/D97*^ *RpS3*^*+/-*^ flies, and sometimes *rpS12*^*D97/+*^ *RpS3*^*+/-*^ flies, were slightly rescued compared to *RpS3*^*+/-*^ flies, but always to a lesser degree than observed with *Xrp1* mutations (Fig 3I–3L). Similar effects of *rpS12* and *Xrp1* mutations were seen on the delayed pupariation of *RpS3*^*+/-*^ larvae (S3 Fig).

### Xrp1-independent regulation of gene expression by RpS12

**Fig 5:**
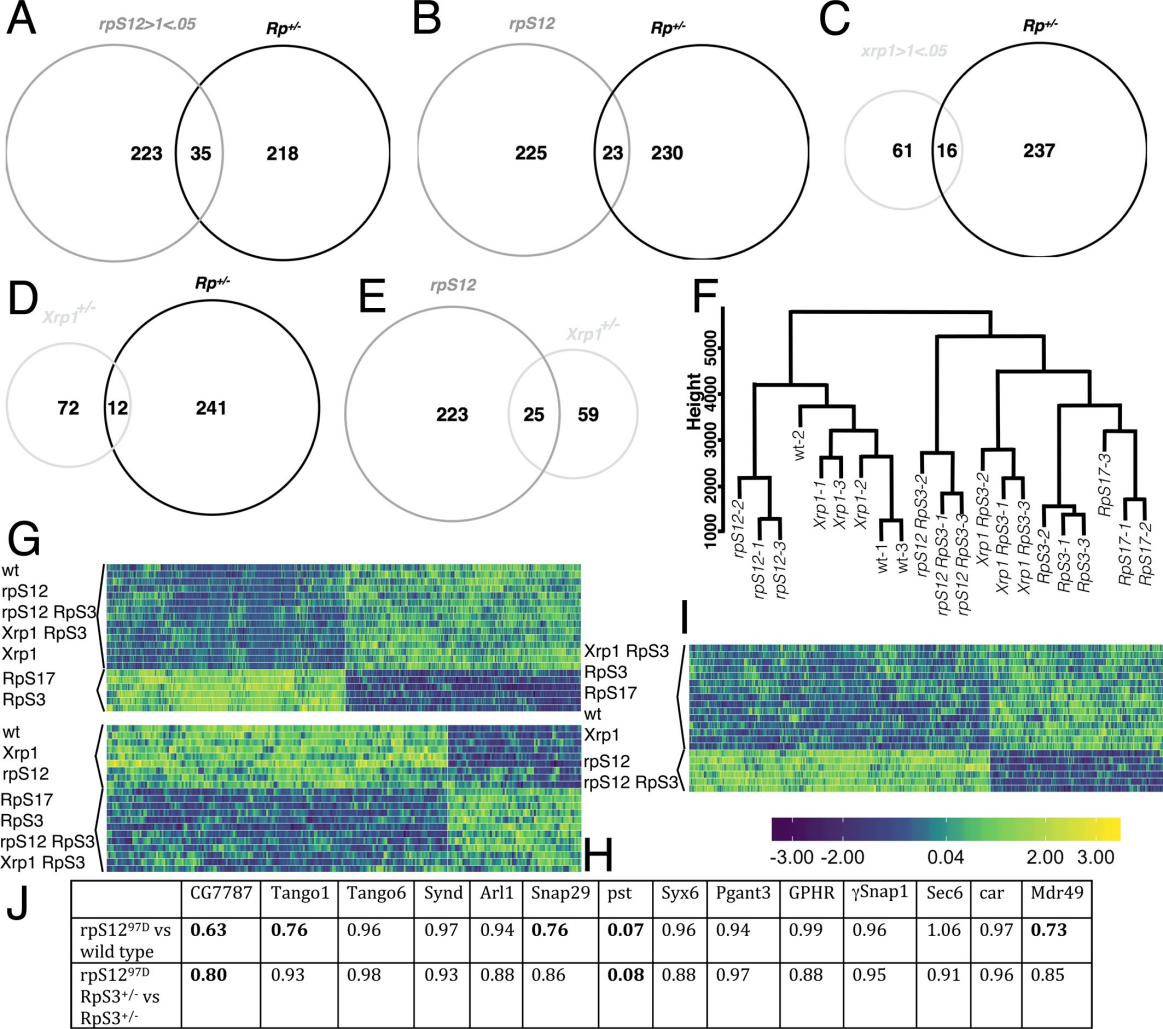
Cross-genotype co-expression analysis. A. Transcripts that differed significantly between control and *rpS12*^*D97/D97*^ wing discs showed little overlap with those affected in *RpS3*^*+/-*^ and *RpS17*^*+/-*^ wing discs. B) Transcripts dependent on RpS12 in both *Rp*^*+/-*^ and *Rp*^*+/+*^ wing discs showed little overlap with those affected in both *RpS3*^*+/-*^ and *RpS17*^*+/-*^ wing discs. C) Transcripts that differed significantly between control and *Xrp1*^*+/-*^ wing discs showed little overlap with those affected in *RpS3*^*+/-*^ and *RpS17*^*+/-*^ wing discs. D) Transcripts dependent on Xrp1 in both *Rp*^*+/-*^ and *Rp*^*+/+*^ wing discs showed little overlap with those affected in both *RpS3*^*+/-*^ and *RpS17*^*+/-*^ wing discs. E) RpS12- and Xrp1-dependent transcripts showed little overlap. F) Hierarchical clustering of wing disc samples by their similarities (as Pearson correlation coefficients) in gene expression profiles distinguished samples by genotypes. The *Xrp1*^*+/-*^ replicates grouped with wild type controls. G) Co-expressed and similarly-regulated genes (module #2) whose expression was altered in *RpS3*^*+/-*^ and *RpS17*^*+/-*^ wing discs in an RpS12-dependent, Xrp1-dependent manner. H) Co-expressed and similarly-regulated genes (module #8) whose expression was altered in *RpS3*^*+/-*^ and *RpS17*^*+/-*^ wing discs in an RpS12-independent, Xrp1-independent manner. I) Co-expressed and similarly-regulated genes (module #6) whose expression was altered in an RpS12-dependent manner regardless of *RpS3* genotype. In G-I, the columns in the heatmaps are genes and the rows are samples (3 replicates each as indicated); color scale indicates relative expression across samples. J) Genes in the Module 6 that were downregulated in *rpS12*^*D97*^ genotypes and present in the GO term ‘secretion’. Their fold changes (from DESeq2) are shown here. Bold values were statistically significant (Padj < 0.05). Neither Module 6 genes that were upregulated in *rpS12*^*D97*^ genotypes, not Module 6 as a whole, was enriched for any particular GO terms.

How the *rpS12*^*D97*^ mutation affects development in *Rp*^*+/+*^ genotypes might be revealed by gene expression studies. DESeq2 identified 258 genes expressed significantly differently in *rpS12*^*D97/D97*^ wing discs compared to wild type, 126 downregulated and 132 upregulated. These genes were significantly enriched for the GO terms related to sensory perception of sweet taste, detection of chemical stimulus involved in the sensory perception of taste, and detection of stimulus involved in sensory perception. Only 35 genes (14%) of these genes were also altered in *Rp*^*+/-*^ wing discs, showing that the *rpS12*^*D97*^ mutation had few effects in common with dominant *Rp* mutations (Fig 5A).

In previous studies it was helpful to focus on *Rp*^*+/-*^ -affected genes whose mRNAs were altered in both *RpS17*^*+/-*^ and *RpS3*^*+/-*^ [24]. We applied a similar cross-comparison to identify RpS12-regulated genes by identifying those whose mRNA levels differed in both *rpS12*^*D97/D97*^ wing discs compared to wild type and in *rpS12*^*D97/D97*^ *RpS3*^*+/-*^ wing discs compared to *RpS3*^*+/-*.^ The 248 genes identified through this cross-comparison were similar to those described above, and only 23 (9%) were also altered in *Rp*^*+/-*^ wing discs (Fig 5B).

DESeq2 identified 77 genes expressed differentially in *Xrp1*^*+/-*^ wing discs compared to wild type, 49 downregulated and 38 upregulated. Alternatively, expression of 84 genes differed in both *Xrp1*^*m2-73/+*^ wing discs compared to wild type and in *Xrp1*^*m2-73/+*^ *RpS3*^*+/-*^ wing discs compared to *RpS3*^*+/-*^. These Xrp1-regulated genes were not enriched for any biological process GO terms, and showed only 21% and 14% overlap, respectively, with genes altered in *Rp*^*+/-*^ wing discs, indicating that *Xrp1* largely failed to regulate the same genes in wild type and in *Rp*^*+/-*^ wing discs (Fig 5C and 5D).

Only 25 RpS12-regulated genes (10%) showed similar regulation by Xrp1 indicating that, in the absence of Minute *Rp* mutations, *rpS12* and *Xrp1* mutations affected largely distinct pathways.

Because the *rpS12*-dependent genes were not obviously enriched for growth genes (in principle genes affecting taste might affect food intake but we would not expect their expression in wing imaginal discs to be responsible), we analyzed gene expression in another way to search for genes and pathways that may be affected in a more subtle way. We performed unsigned Weighted Gene Co-expression Network Analysis (WGCNA) to identify sets of genes with similar co-expression patterns, referred as gene modules. Importantly, WGCNA can identify changes in gene expression that may not reach the statistical threshold for detecting differential expression of individual genes and allow integrated analysis of samples across multiple genotypes. Clustering analysis of samples by their gene expression similarities showed that samples of different genotype clustered separately, except that *Xrp1*^*+/-*^ samples were intermingled with the wild type control, suggesting that in non-Minute genotypes *Xrp1* has only a minimal effect on mRNA abundance (Fig 5F). WGCNA analysis of the 21 samples identified 20 gene modules, containing various numbers of genes with significantly positively or negatively correlated expression patterns. Of these, Module #2 contained genes elevated in both *RpS3*^*+/-*^ and *RpS17*^*+/-*^, in an RpS12/Xrp1-dependent manner (Fig 5G). Interestingly, Module #2 contained many of the RpS12/Xrp1-regulated genes already identified as differential expression in Minute *Rp* mutants. Module #8 contained genes elevated in both *RpS3*^*+/-*^ and *RpS17*^*+/-*^, in an RpS12/Xrp1-independent manner (Fig 5H). Module #8 was enriched for GO terms associated with cytoplasmic translation, indicating that Minute *Rp* mutants may also affect translation independently of RpS12/Xrp1. Module #6 contained RpS12-regulated genes (altered in *rpS12*^*D97/D97*^ *RpS3*^*+/-*^ compared to *RpS3*^*+/-*^ and in *rpS12*^*D97/D97*^ compared to wild type) (Fig 5I). The Module #6 genes that were down-regulated in *rpS12*^*D97*^ genotypes were enriched for genes involved in protein secretion, although with the exception of pastrel *(pst*) the effects on gene expression were minor (Fig 5J). We did not detect any module of genes clearly regulated by Xrp1 alone.

Although these experiments did not identify how the *rpS12*^*D97*^ mutation might affect growth independently of Xrp1, they confirmed that individually, *Rp*^*+/-*^, *rpS12*^*D97*^ and *Xrp1*^*+/-*^ mutants had little in common, suggesting that the RpS12-Xrp1 regulatory axis is not active in non-Minute wing discs. Indeed, gene expression in Xrp1^+/-^ wing discs was indistinguishable from the controls by WGCNA analysis (Fig 5F).

### Regulation of *Rp* gene transcripts

**Fig 6:**
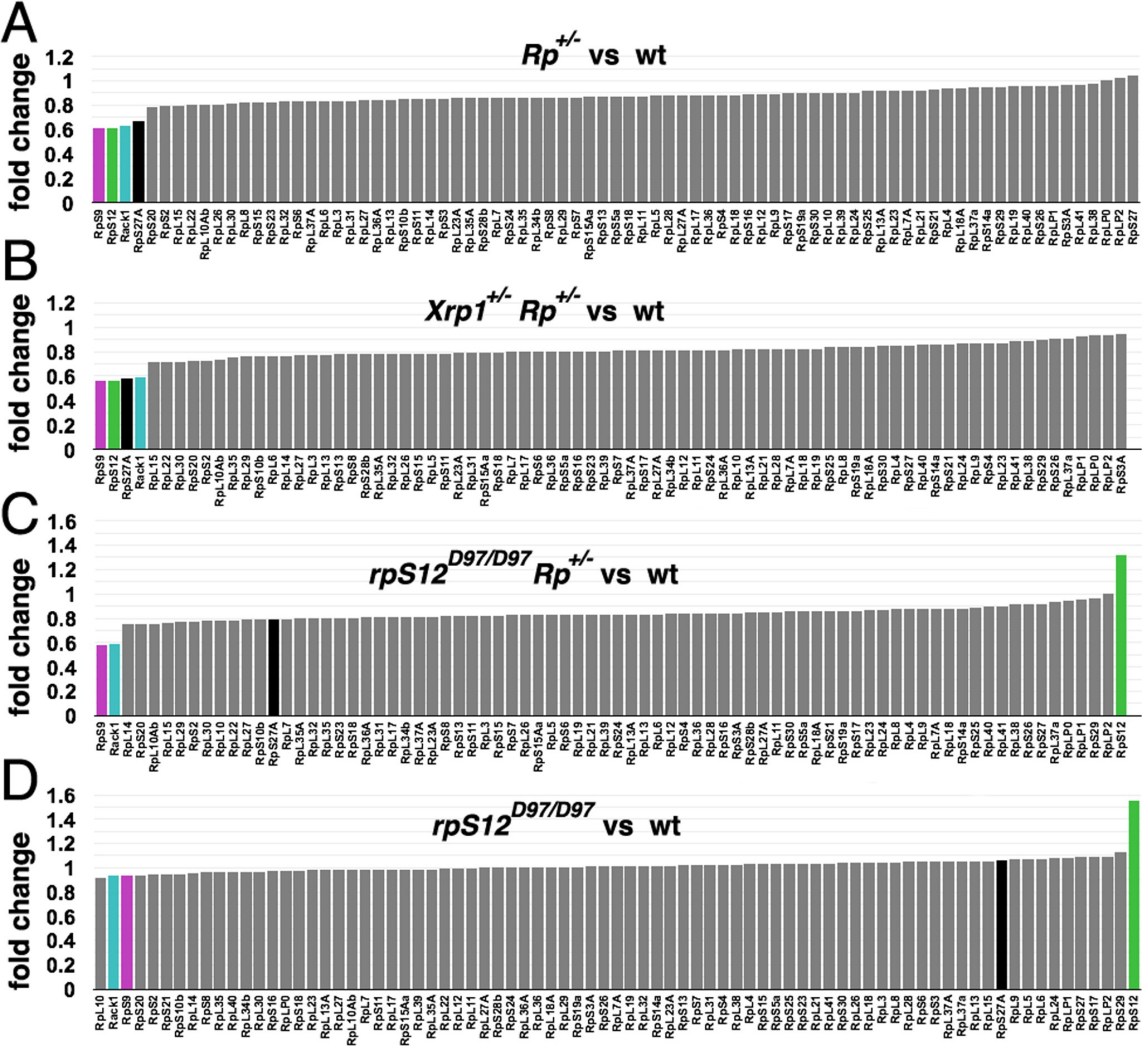
Ribosomal protein transcripts. All panels show fold changes of *Rp* mRNA levels from mRNA-seq analysis of mutant wing imaginal discs in comparison to wild type controls. Loci are arranged from most reduced to most increased in each case. Transcripts of duplicated *Rp* genes that are expressed in at low levels because their paralog dominates in wing discs are not included. *RpS9*, *rpS12*, *RACK1*, *RpS27A* transcripts highlighted. A) *Rp* genes show an overall reduction in transcript levels in *RpS3*^*+/-*^ and *RpS17*^*+/-*^ wing discs, with *RpS9*, *rpS12*, *Rack1*, and *RpS27A* affected to a greater extent. Fold changes are the mean of *RpS3*^*+/-*^ and *RpS17*^*+/-*^ values except for *RpS3* and *RpS17* themselves; transcript levels of these genes is 50% reduced in their own mutants so only the value for the other mutant is shown here. B) *Rp* mRNAs in *RpS3*^*+/-*^ *Xrp1*^*+/-*^ wing discs resembled those in *RpS3*^*+/-*^ wing discs. *RpS3* mRNA levels are not included. C) *RpS9* and *Rack1* transcript levels remained reduced in *rpS12*^*D97/D97*^ *RpS3*^*+/-*^ but *rpS12* and *RpS27A* were restored to wild type or higher levels. *RpS3* mRNA levels are not included. D) The only *Rp* whose transcript levels were affected in *rpS12*^*D97/D97*^ wing discs was *rpS12* itself.

The GO term ‘cytoplasmic translation’ was enriched among transcripts that were affected by Minute *Rp* mutants independently of RpS12 and Xrp1 (Module 8: Fig 5H). This reflects presence of many *Rp* gene transcripts in this module. We therefore looked at the transcript levels of all *Rp* genes in wing discs (Fig 6). *Rp* transcripts are highly expressed (>36% of total transcripts in wild type wing discs). Both *RpS3*^*+/-*^ and *RpS17*^*+/-*^ led to a general reduction in mRNA levels of *Rp* genes (median fold change -13%). In addition, *RpS9*, *RpS12*, *Rack1* and RpS27A genes stood out as being more strongly reduced (Fig 6A). This picture changed very little in *RpS3*^*+/-*^ *Xrp1*^*+/-*^ wing discs, indicating that these changes in *Rp* expression were independent of *Xrp1*(Fig 6B). In *RpS3*^*+/-*^ *rpS12*^*D97/D97*^ wing discs, however, only *RpS9* and *Rack1* were still significantly lower than the general distribution of *Rp* transcripts (Fig 6C). Thus, reduction in *RpS27A* mRNA levels in *RpS3*^*+/-*^ discs was a specific function of *rpS12*. The *rpS12* mRNA itself was significantly elevated in *RpS3*^*+/-*^ *rpS12*^*D97/D97*^ wing discs(Fig 6C). This could reflect a role of RpS12 in suppressing its own expression, as revealed when *rpS12*^*D97/D97*^ wing discs were compared to controls. In this case all other *Rp* mRNA levels were at control levels (median fold change 1%), but *RpS12* levels were elevated by 55% (Fig 6D).

These findings identify a hierarchy of *Rp* transcript regulation. Both *RpS3*^*+/-*^ and *RpS17*^*+/-*^ genotypes modesty reduce mRNA levels of all *Rp* genes in an RpS12-Xrp1-independent manner (median fold change 13–20% in the various genotypes). The reductions of *RpS9* and *Rack1* levels stand out as still more extreme (-39% and -37% fold change, respectively). *RpS27A* and *rpS12* are also reduced, but in an RpS12-dependent manner that does not depend on *Xrp1*. These findings illustrate that not all aspects of the *Rp*^*+/-*^ phenotype depend on *rpS12* or *Xrp1*. Since it is *Xrp1* that is responsible for much of the reduction in bulk translation rate that occurs in ‘Minute’ *Rp*^*+/-*^ genotypes [24], how much these modest, Xrp1-independent changes in *Rp* transcripts affect steady-state ribosome number or overall translation is uncertain.

### Evidence for an RpS12-independent contribution of Xrp1 to the rate of development

**Fig 7:**
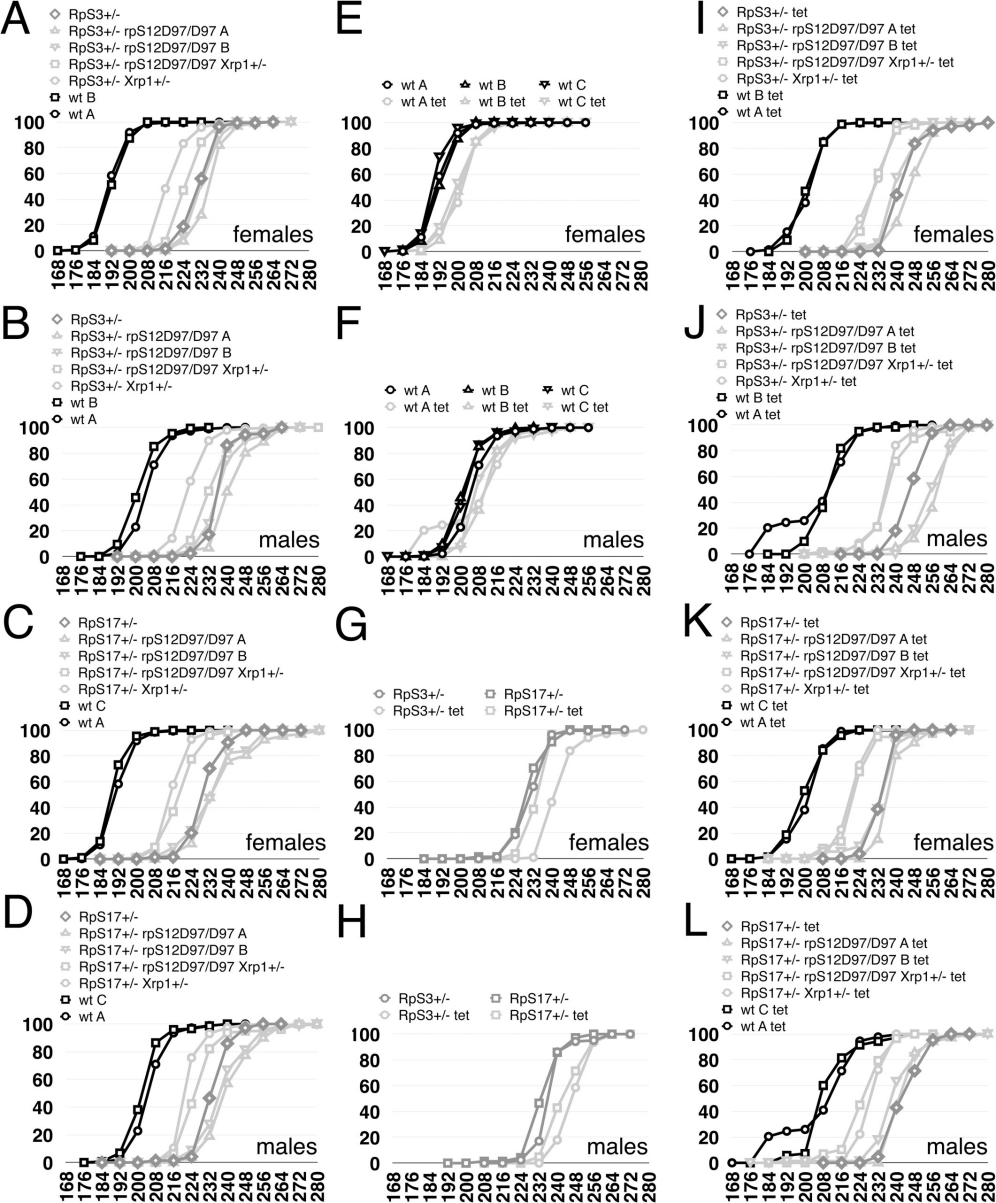
Effects of Xrp1 and of tetracycline on rate of development. All panels show the cumulative percentage of adult flies emerged according to time after egg laying in hours. A,B) An experiment in which *rpS12*^*D97/D97*^ and *rpS12*^*D97/+*^ genotypes did not accelerate the development of *RpS3*^*+/-*^ flies, and in which *RpS3*^*+/-*^ *rpS12*^*D97/D97*^ *Xrp1*^*+/-*^ flies developed more slowly than *RpS3*^*+/-*^ *Xrp1*^*+/-*^ flies. C,D) An experiment in which *rpS12*^*D97/D97*^ and *rpS12*^*D97/+*^ genotypes did not accelerate the development of *RpS17*^*+/-*^ flies, but in which *RpS17*^*+/-*^ *rpS12*^*D97/D97*^ *Xrp1*^*+/-*^ flies developed as rapidly as *RpS17*^*+/-*^ *Xrp1*^*+/-*^ flies. E,F) Tetracyclin feeding modestly retarded development of wild type flies. G,H) Tetracyclin feeding modestly retarded development of *RpS3*^*+/-*^ and *RpS17*^*+/-*^ flies. I,J) After tetracycline feeding, *rpS12*^*D97/D97*^ and *rpS12*^*D97/+*^ genotypes did not accelerate the development of *RpS3*^*+/-*^ flies, but in which *RpS3*^*+/-*^ *rpS12*^*D97/D97*^ *Xrp1*^*+/-*^ flies developed as rapidly as *RpS3*^*+/-*^ *Xrp1*^*+/-*^ flies. K,L) After tetracycline feeding, *rpS12*^*D97/D97*^ and *rpS12*^*D97/+*^ genotypes did not accelerate the development of *RpS3*^*+/-*^ flies, but in which *RpS3*^*+/-*^ *rpS12*^*D97/D97*^ *Xrp1*^*+/-*^ flies developed as rapidly as *RpS3*^*+/-*^ *Xrp1*^*+/-*^ flies. The genotypes used and numerical data corresponding to these graphs is tabulated in the S8 Table.

To help understand how RpS12 and Xrp1 function together, developmental delay was assessed in the triple mutant combination *rpS12*^*D97/D97*^ *RpS3*^*+/-*^ *Xrp1*^*+/-*^. If the hypothesis that RpS12 acts through Xrp1 is correct, we would expect that Xrp1 would make no contribution to the developmental delay of *rpS12*^*D97/D97*^ *RpS3*^*+/-*^ flies, in which *Xrp1* expression is not elevated, so that *rpS12*^*D97/D97*^ *RpS3*^*+/-*^ *Xrp1*^*+/-*^ and *rpS12*^*D97/D97*^ *RpS3*^*+/-*^ would develop similarly. In the experiment described in a previous section, where *rpS12*^*D97/D97*^ *RpS3*^*+/-*^partially suppressed the developmental delay of *RpS3*^*+/-*^, a similar or greater suppression was seen with *rpS12*^*D97/D97*^ *RpS3*^*+/-*^ *Xrp1*^*+/-*^ (Fig 3K and 3L). We do not always, however, see suppression of developmental delay by *rpS12*^*D97*^ [23]. In an independent experiment where *rpS12*^*D97/D97*^ *RpS3*^*+/-*^ and *rpS12*^*D97/D97*^ *RpS17*^*+/-*^ were not rescued in comparison to *RpS3*^*+/-*^ *and RpS17*^*+/-*^, developmental delay was partially suppressed in *rpS12*^*D97/D97*^ *RpS3*^*+/-*^ *Xrp1*^*+/-*^ and suppressed in *rpS12*^*D97/D97*^ *RpS17*^*+/-*^ *Xrp1*^*+/-*^ to the same degree as in *RpS17*^*+/-*^ *Xrp1*^*+/-*^ (Fig 7A–7D). The variable phenotype of *rpS12*^*D97*^ *Rp*^*+/-*^ genotypes is unexpected. We do not think this reflects inaccuracy in the measurement method. The rescue observed in Fig 3K and 3L was highly significant statistically and also observed when timing of pupariation was measured (S3 Fig). The various panels of Fig 3 and Fig 7 together contained 34 internal controls of independently replicated measurements (eg of multiple wild type genotypes) that illustrate very high reproducibility for other genotypes. It is possible that *rpS12*^*D97*^ *Rp*^*+/-*^ genotypes behave differently because their rate of development arises as a balance of multiple positive and negative processes.

We considered the possibility that microbiome differences might affect our results. Cultures of *Rp*^*+/-*^ genotypes can grow poorly and may be more prone to microbial growth, which can be suppressed by supplementing fly food with antibiotics. Interestingly, tetracycline measurably delayed development of all genotypes, including wild type controls (eg Fig 7E–7H). Whether this reflects a positive contribution of the microbiome to fly growth, or a deleterious effect of tetracycline [27, 28] is uncertain. In the presence of antibiotic, *rpS12*^*D97/D97*^ *RpS3*^*+/-*^and *rpS12*^*D97/D97*^ *RpS17*^*+/-*^developed no faster than *RpS3*^*+/-*^or *RpS17*^*+/-*^, but *rpS12*^*D97/D97*^ *RpS3*^*+/-*^ *Xrp1*^*+/-*^, *RpS3*^*+/-*^ *Xrp1*^*+/-*^, *rpS12*^*D97/D97*^ *RpS17*^*+/-*^ *Xrp1*^*+/-*^ and *RpS17*^*+/-*^ *Xrp1*^*+/-*^ all showed a similar acceleration of development compared to *RpS3*^*+/-*^and *RpS17*^*+/-*^(Fig 7I–7L). Therefore, although the results are complicated by variability of *rpS12*^*D97*^ *Rp*^*+/-*^ genotypes, *Xrp1* mutation frequently accelerated the development of *rpS12*^*D97/D97*^ *RpS3*^*+/-*^ flies, despite the fact that *Xrp1* expression was not elevated in *rpS12*^*D97/D97*^ *RpS3*^*+/-*^ wing discs.

## Discussion

**Fig 8:**
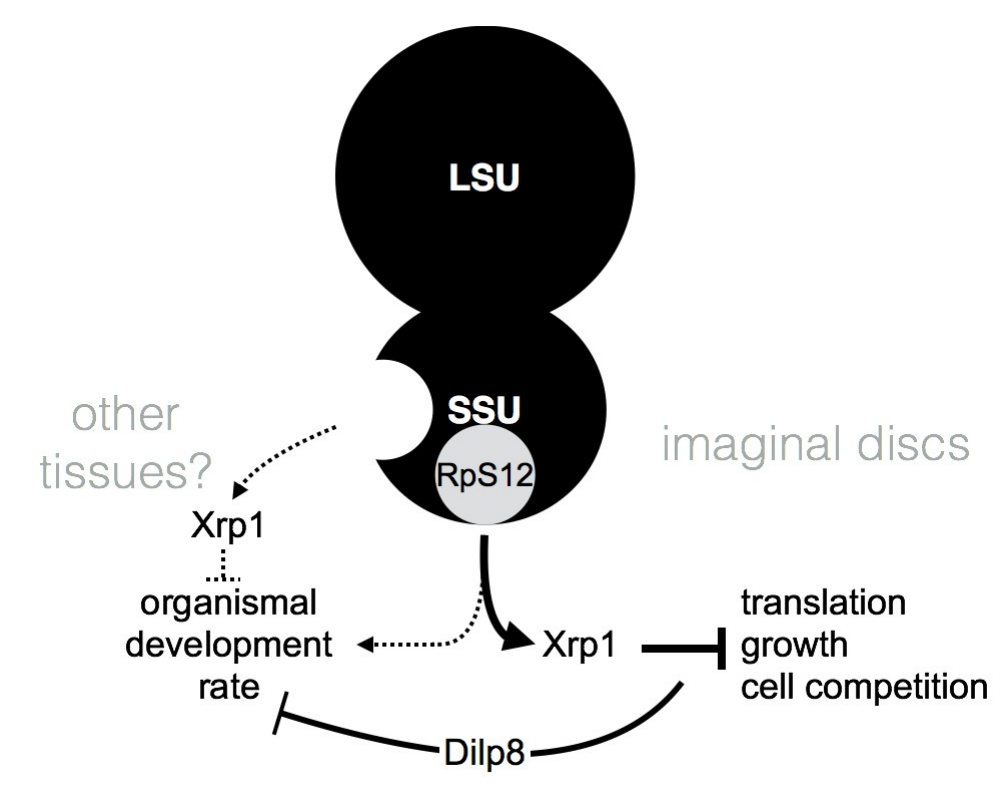
Model for regulatory effects of *Rp* mutations. When one of the many haploinsufficient Rp is limiting, an RpS12-dependent signal is activated that elevates Xrp1 expression, inhibiting imaginal disc cell translation and growth and making *Rp*^*+/-*^ cells less competitive. Analyses of imaginal disc gene expression and genetic epistasis studies provide compelling evidence that RpS12 acts through Xrp1 in imaginal discs. Defects in imaginal disc growth delay overall organism development, which may depend on Xrp1-dependent signaling by Dilp8 [21]. Other data suggest that RpS12 also contributes positively to the rate of development, independently of Minute *Rp* mutations, and that Minute *Rp* mutations and Xrp1 may also slow organism development independently of RpS12.

Our findings provide compelling evidence that RpS12 contributes to the ‘Minute’ *Rp*^*+/-*^ phenotype through Xrp1. In contrast to initial impressions that RpS12 might play a more specific role in cell competition than Xrp1, because *rpS12* mutations seemed to suppress less of the ‘Minute’ *Rp*^*+/-*^ phenotype, it is in fact RpS12 that is the more upstream of the two cell competition genes (Fig 8).

The RpS12-dependent and Xrp1-dependent components of the *Rp*^*+/-*^ gene expression signature overlap almost completely, indicating that they affect the same pathway (Fig 1A–1C). These RNA-Seq results also suggest that RpS12 acts upstream of Xrp1. They show that Xrp1 expression is RpS12-dependent; conversely, Xrp1 makes no contribution to RpS12 expression in *Rp*^*+/-*^ genotypes. There are also transcripts, such as *RpS27A*, that are altered in *Rp*^*+/-*^ wing discs in a way that depends on RpS12 but not on Xrp1 (Fig 1C, Fig 6).

Previously we had noted that RpS12 is required for the elevated Xrp1 expression seen in *Rp*^*+/-*^ cells [16]. Here we confirm this regulation is cell-autonomous, and importantly we show, through genetic epistasis, that Xrp1 is indeed required downstream of RpS12 in cell competition, as the regulation of Xrp1 expression levels would suggest (Fig 2A–2D). In addition we showed that, as would be expected if RpS12 acts through Xrp1, the *rpS12*^*D97*^ mutation also prevents the reduction in overall translation rate that Xrp1 expression causes in ‘Minute’ *Rp*^*+/-*^ wing discs (Fig 2E–2L).

These molecular and genetic findings demonstrate clearly that RpS12 not only affects Xrp1 expression but that through Xrp1 it is required for almost the entire gene expression program of *Rp*^*+/-*^ wing discs as well as their growth and global translation rate. The close correspondence between the *Xrp1* and the *rpS12*^*D97*^ mutations argues that Xrp1 is the main target of RpS12 in *Rp*^*+/-*^ wing discs. Because RpS12 is a ribosomal protein, it may be the signal, or part of the signal, that communicates a failure of ribosome assembly to the cell (Fig 8) [23]. While the signaling mechanism is not yet known, genetic studies indicate that the cell-competition mutation *rpS12*^*D97*^ affects a different aspect of RpS12 function from the essential function that reflects the role of RpS12 in general translation (which is also not yet understood) [23], and here we report accordingly that the gene expression changes caused by the *rpS12*^*D97*^ mutations are largely unrelated to those caused by mutations in most other *Rp* genes (Fig 5). Many ribosomal proteins, all of which are very highly expressed proteins, have a minor pool that participates in extra-ribosomal activities [2], but it also cannot be excluded that RpS12 might make two distinct contributions to ribosome function, only one of which is affected by *rpS12*^*D97*^ [23].

The role of RpS12 as an upstream regulator of Xrp1 was surprising at first, because *rpS12*^*D97*^ had not seemed to suppress the developmental delay of ‘Minute’ *Rp*^*+/-*^ genotypes as *Xrp1* mutations did [23]. If Xrp1 expression causes the developmental delay, and RpS12 is required for elevated Xrp1 expression, then RpS12 should be required for the developmental delay. Several new lines of evidence suggest that this is the case. First, we found that extra copies of RpS12 enhanced the developmental delay of *RpS3*^*+/-*^, suggesting that wild type RpS12 does promote developmental delay (Fig 3A and 3B). Secondly, we now find multiple examples where *rpS12* mutations do suppress the developmental delay of Minutes, albeit always to a lesser extent than *Xrp1* mutations did (Fig 3C, 3D, 3I–3L).

A potential explanation why the *rpS12*^*D97*^ mutation has less effect on the developmental delay of *Rp*^*+/-*^ genotypes than *Xrp1*, and sometimes no effect at all, could be that *rpS12*^*D97*^ has other deleterious effects on development, so that in *rpS12*^*D97/D97*^ *Rp*^*+/-*^ genotypes the negative effects of the *rpS12*^*D97*^ mutation partially or completely cancel out the effect of reduced Xrp1 expression. Several lines of evidence are now consistent with this hypothesis. First, a modest but consistent delay of development is observed in *rpS12*^*D97/*D97^ and *rpS12*^*D97/-*^ genotypes in the absence of other *Rp* mutations (Fig 3E–3H). Secondly, *rpS12*^*D97/*D97^ and rpS12^D97/-^ exhibit reduced longevity and reduced adult wing size, consistent with a deleterious effect even in the absence of other *Rp* mutations (Fig 4). We did not, however, identify any candidate growth pathway from gene expression studies of *rpS12*^*D97/*D97^ wing discs, which seems to share little in common with the effects of mutations at haploinsufficient loci such as *RpS3* and *RpS17* (Fig 5A, 5B, 5F and 5J). Given that *rpS12*^*D97/*D97^ mutant clones grow at the same rate in wing discs as wild type controls [23], the possibility exists that the *rpS12*^*D97*^mutation might affect developmental growth rate through another tissue, in which case these effects may not be not reflected in wing disc mRNA sequencing results.

A recent study has also implicated RpS12 and Xrp1 in the systemic delay of organismal development that results from damaging the wing disc by *Rp* knockdown. The wing disc damage induces organismal delay through RpS12- and Xrp1-dependent regulation of Dilp8, a further example where RpS12 signals through Xrp1 [21]. *Rp*^*+/-*^ genotypes, although probably less severe than *Rp* knockdown, also elevate *dilp8* transcription (Fig 1C and [16]), so there could also be a systemic component to the ‘Minute’ *Rp*^*+/-*^ developmental delay. If the overall developmental rate of ‘Minute’ *Rp*^*+/-*^ flies is determined by interactions between multiple tissues, not just the rate of imaginal disc growth alone, this would be consistent with some of the effects of RpS12 and Xrp1 could occur in other tissues.

Because *rpS12*^*D97/*D97^ flies are unable to eliminate *Rp*^*+/-*^ cells by cell competition, defects in these flies could also reflect positive roles for cell competition in healthy aging. This has been suggested based on reduced longevity of and accumulation of developmental defects in *azot* mutants, encoding another gene implicated in cell competition [29]. It is notable, however, that loss of *Xrp1* does not affect longevity [18], and we do not see developmental defects in either *rpS12*^*D97/*D97^ or *Xrp1*^*+/-*^ flies, suggesting that these aspects of the *azot* phenotype are not due to failure to eliminate *Rp*^*+/-*^ cells, although they could reflect failure to eliminate other kinds of cells.

*Xrp1* appears to slow growth cell-autonomously by reducing overall translation [24]. In *Rp*^*+/-*^ genotypes, *Xrp1* is responsible for regulating three regulators of cytoplasmic ribosome assembly, *eIF6*, *CG8549* and *CG33158* (Fig 1A). *CG8549* encodes the *Drosophila* homolog of the Shwachman-Diamond syndrome protein. In humans and in yeast these genes provide a quality control mechanism at the final step in LSU assembly that functionally activates the 60S subunit [30, 31], although eIF6 also promotes growth through other mechanisms [32]. These genes are *up*-regulated by Xrp1 in *Rp*^*+/-*^ wing discs, however, which should not make them limiting for ribosome biogenesis or growth (Fig 1A). The *Rp*^*+/-*^ genotypes also show a modest (~15%) reduction in mRNA levels of most Rp genes, but this was Xrp1-independent, suggesting that this is not the mechanism reducing translation in *Rp*^*+/-*^ genotypes, although it could contribute to Xrp1-independent growth reduction in *Rp*^*+/-*^ genotypes(Fig 6).

The RpS12-Xrp1 axis adds to the notion that many effects of *Rp* mutations are due to regulatory changes, not direct effects of a hypothetical reduction in ribosome numbers [16]. A regulatory pathway of nucleolar stress is also thought to contribute to Diamond-Blackfan Anemia, the human disorder associated with heterozygous mutations in *Rp* genes, and might contribute to the properties of human tumors with *Rp* mutations. In mammalian cells where ribosome assembly is reduced, unassembled RpL5 and RpL11 stabilize p53 [33, 34]. It is not yet clear how much p53 contributes to the phenotype, for example it is not yet established whether p53 is responsible for reducing translation, and it is debated whether p53 is responsible for anemia [35–39]. How mammalian RpS12 might be involved in Diambond Blackfan Anemia or cancer has not been described, although a possible association has been reported between RpS12 deletion and Diffuse Large B Cell Lymphoma [7, 40].

## Methods

### Experimental animals

Species: *Drosophila melanogaster*. Strains were generally maintained at 25°C on medium containing the following ingredients per 1L: 18g yeast; 22g molasses; 80g malt extract; 9g agar; 65g cornmeal; 2.3g methyl para-benzoic acid; 6.35ml propionic acid unless otherwise indicated. Sex of larvae dissected for most imaginal disc studies was not differentiated.

### Fly stocks

Transgenes strains used in this study included: P{*arm-LacZ*, *w+}* [41], P{*ubi-GFP*, *w+}* [42]; P{rpS12^+^-8kb, w+} [23]. Mutant alleles used in this study: *rpS12*^*D97*^ [23], *Xrp1*^*M2-73*^ [13], *M(2)56F* (laboratory of Y. Hiromi), *RpS17*^*4*^ [10], *RpS3* [43].

### Clonal analysis

Genetic mosaics were generated using the FLP/FRT system employing hsFLP and eyFLP transgenic strains [44–46]. For making clones using inducible *hsFLP*, larvae of non-Minute genotypes were subjected to 1 hour heat shock at 37°C, 60 ± 12 hours after egg laying and dissected 60hr later. For Minute/+ genotypes, heat shock was administered after 84 ± 12 hours of egg laying and dissected 72 hours later. Detailed genotypes employed are listed in figure legends.

### Measurement of developmental timing

For measurements of developmental rate, adults were allowed to lay eggs on yeast-glucose media changed at 8h intervals, with or without tetracycline at 20 μg/ml final concentration [47, 48]. Multiple cultures were established in parallel to generate sufficient numbers, and typically maintained for 7–10 days of passage every 8h. Overcrowded or near-barren vials were discarded. Adults were collected every 8h to record emergence time. Whenever possible, genotypes emerging in the same cultures were compared and in other cases, control genotypes that were also present could be compared between crosses to verify comparable conditions (see the S4 Table & S8 Table). In most experiments shown, multiple independent estimates were obtained for the developmental rate of some genotypes within these crosses. These were very similar in every case, verifying the reproducibility of the assays (see Figs 3 & 7). For some experiments, pupariation was also recorded at 8h intervals.

### Immunohistochemistry and antibody labeling

For antibody labeling, imaginal discs were dissected from late 3rd instar larvae, fixed and processed for immunohistochemistry as described previously [49]. Primary Antibodies are described in the Key Resources Table. Secondary Antibodies were Cy2-, Cy3- and Cy5- conjugates from Jackson Immunoresearch.

### Image acquisition and processing

Confocal images were recorded using Leica SP2, SP5 and SP8 confocal microscopes using 20x and 40x objectives. Images were processed using Image J1.44j and Adobe Photoshop CS5 Extended.

### Measurement of translation in vivo

Translation was detected by the Click-iT Plus OPP Alexa Fluor 594 or 488 Protein Synthesis Assay Kits (Thermofisher) as described [50] with modifications as described [24]. OPP (*o*-propargyl puromycin) is a puromycin analog that is incorporated into nascent polypeptide chains during a 15 min incubation with explanted imaginal discs.

### mRNA-Seq

These mRNA-Seq analyses described here were performed in parallel to studies of other genotypes described previously, using the same methods [24]. Precise genotypes for which data are described for the first time were:

*w*^*11-18*^; *rpS12*^*D97*^ *FRT80B / rpS12*^*D97*^ *FRT80B*
*w*^*11-18*^; *rpS12*^*D97*^ *FRT80B* / *rpS12*^*D97*^ *FRT82B RpS3*
*w*^*11-18*^; *FRT82B Xrp1*^*m2-73*^ */ +*

At least 65,000,000 clean RNA-seq reads past quality controls were obtained from every sample and for every sample at least 89% of them were mapped to the fly genome. Bioconductor DESeq2 [51] was used to identify genes expressed significantly differently between control and *rpS12*^*D97/D97*^ wing discs and between control and *Xrp1*^*m2-73/+*^ wing discs, using the criteria log2(fold change)>1, adjusted p-value (Padj)<0.05. We also exploited cross genotype comparisons to define RpS12-regulated genes as those that were expressed differentially from control in *rpS12*^*D97/D97*^ and also expressed differently from RpS3^+/-^ in *rpS12*^*D97/D97*^ *RpS3*^*+/-*^, with |log_2_fold change|>0.5 and Padj<0.1 in both cases, and to define Xrp1-regulated genes as those that were expressed differentially from control in *Xrp1*^*+/-*^ and expressed differently from *RpS3*^*+/-*^ in *Xrp1*^*+/-*^ *RpS3*^*+/-*^, with |log_2_fold change|>0.5 and Padj<0.1 in both cases. GO enrichment analysis was performed using Gene Ontology Consortium tool PANTHER [52–54]. We considered GO terms to be significantly enriched when p<0.05 after Benjamini correction for multiple tests. This correction contributes to some differences in enriched terms in our analyses compared to those from another group [22].

### Gene co-expression network

A total of 8,321 expressed genes, with FPKM (Fragments Per Kilobase of transcript per Million mapped read) > 0 in at least one sample, were used for weighted gene correlation network analysis (WGCNA) [55, 56]. We transferred the FPKM values into Transcripts Per Million (TPM) and utilized “normalizeBetweenArrays” function in the “limma” package [57] to normalize the TPM values across samples. We chose the power threshold of 9 and minimal module size > 30, and obtained 20 gene modules, with sizes ranging from 1,299 to 48 genes. Since we used the parameter “unsigned” in computing correlation in order to maximize the gene co-expression network, the expression of a module gene could be either positively or negatively correlated to the module eigengene (the first principal component of each gene module).

### Quantification and statistical analyses

Statistical comparisons between individual mutant and control experiments were by Student’s t-test or by the Mann-Whitney procedure, as indicated in Figure Legends. Significance of differential gene expression derived from mRNA-Seq results was the adjusted P value determined by DESEQ2.

## Supporting information

S1 Table Fold changes of mRNAs expressed differentially in *Rp*^*+/-*^ wing discs. Fold changes (determined by DESeq2) in mRNA levels between wing discs from wild type and from indicated genotypes. Significant differences (Padj<0.05) indicated in bold. Genes shown here include all those corresponding to the enriched GO terms mature ribosome assembly (R), sulfur compound metabolic process (S), glutathione metabolic process (G), telomere maintenance (T), and DNA repair (D). These data correspond to the heatmap in Fig 1C.
(PDF)

S2 Table Effects of *RpS3* and *RpS17* mutations on targets of major signaling pathways. Fold changes (determined by DESeq2) in mRNA levels between wing discs from wild type and from indicated genotypes. Significant differences (Padj<0.05) indicated in bold.
(PDF)

S3 Table Transcription factor motif enrichment in *Rp*^*+/-*^-regulated genes. (PDF)

S4 Table Numerical data corresponding to Fig 3A–3L. (XLSX)

S5 Table Numerical data corresponding to S3 Fig. (XLSX)

S6 Table Survival data corresponding to Fig 4A and 4B and S4 Fig. (XLSX)

S7 Table Wing size data corresponding to Fig 4C. (XLSX)

S8 Table Numerical data corresponding to Fig 7A–7L. (XLSX)

S1 Fig Further examples of translation in RpS17^+/-^ wing discs. Comparing translation rates measured by OPP incorporation between different regions of wing discs is complicated to some extent by the dynamic patterns of translation that occur the in wild type [16], perhaps reflecting the dynamic and patchy activity of TORC1 that is revealed by RpS6 phosphorylation patterns[58]. Changes due to mutations in *Rp* genes are superimposed upon this variable background and are best assessed by directly observing how translation changes cell-autonomously along sharp clone boundaries. In support of the conclusion that *RpS17* mutations reduce translation in an RpS12-dependent manner (see Fig 2E–2H), we present additional examples of cell autonomous differences in translation rate between *RpS17*^*+/-*^ cells, labeled with GFP, and unlabeled *RpS17*^*+/+*^ clones (panels A-G). Translation, shown by OPP incorporation in panels A’-G’, is consistently lower in *RpS17*^*+/-*^ regions. In contrast to these *rpS12*^*+/+*^examples, clones of *RpS17*^*+/+*^ *rpS12*^*D97/D97*^cells in *RpS17*^*+/-*^ *rpS12*^*D97/D97*^wing discs (panels H-L) show no difference in translation rate (panels H’-L’).
(TIF)

S2 Fig Cell-autonomous translation effects of RpS12 in Rp^+/-^ wing discs. Panels A-F show wing discs containing clones of indicated genotypes. Corresponding translation rate (OPP incorporation) is shown in panels A’-F”, and the overlay of translation and genotype in panels A”-F”. A,B,C indicated that *rpS12* had cell-autonomous, Xrp1-dependent effects on translation in *RpS3*^*+/-*^ cells. (A-A” and B-B”) *RpS3*^*+/-*^ *rpS12*^*+/+*^ clones often show lower translation than the *RpS3*^*+/-*^ *rpS12*^*D97/+*^ background (eg orange arrows). Translation was often higher in *RpS3*^*+/-*^ *rpS12*^*D97/D97*^ clones (eg yellow arrows). (C-C”). In the *Xrp1*^*+/-*^ background, translation rates were unaffected by *rpS12* genotype. D,E,F show *RpS17*^*+/-*^ genotypes. Because *rpS12* and *RpS17* both map to chromosome 3L, FLP-mediated recombination cannot generate *RpS17*^*+/-*^ *rpS12*^*D97/D97*^ and *RpS17*^*+/-*^ *rpS12*^*+/+*^ clones in the same disc. These figures show that, unlike *RpS17*^*+/-*^ *rpS12*^*+/+*^ cells (see main text Fig 2E and 2F), translation in the *RpS17*^*+/-*^ *rpS12*^*D97/+*^ genotype was only sometimes distinguishable from that of *RpS17*^*+/+*^ cells. (D-D”). In some cases, *RpS17*^*+/+*^ *rpS12*^*+/+*^ clones showed translation rates higher than the *RpS17*^*+/-*^ *rpS12*^*D97/+*^ background (eg cyan arrows). (E-E”). In most cases, *RpS17*^*+/+*^ *rpS12*^*+/+*^ clones typically showed translation rates similar to the *RpS17*^*+/-*^ *rpS12*^*D97/+*^ background (eg cyan arrows). (F-F”) Little or no translation difference was seen between *RpS17*^*+/+*^ *rpS12*^*D97/D97*^ clones and the *RpS17*^*+/-*^ *rpS12*^*D97/+*^ background (eg blue arrows). Genotypes used. A,B) y w hsF; rpS12^97D^ FRT80B M(3)95A /rpS12^97D^ FRT80B P{arm-LacZ}. C) y w hsF; rpS12^97D^ FRT80B M(3)95A/rpS12^97D^ FRT80B P{arm-LacZ} Xrp1^m2-73^. D,E) y w hsF; RpS17^4^ rpS12^97D^ P{ubi-GFP} FRT80B/FRT80B. F) y w hsF; RpS17^4^ P{ubi-GFP} FRT80B/rpS12^97D^ FRT80B.
(TIF)

S3 Fig Contribution of RpS12 to the timing of pupariation. The timing of pupariation was measured in the same experiment shown in Fig 3K and 3L. Like the effects on adult emergence, *rpS12*^*D97/D97*^ and *rpS12*^*D97/+*^ genotypes suppressed the delay to pupariation on *RpS3*^*+/-*^ larvae, to a similar extent to *RpS3*^*+/-*^ *rpS12*^*D97/D97*^ *Xrp1*^*+/-*^ larvae. The detailed genotypes used and numerical data corresponding to these graphs is tabulated in S5 Table.
(TIF)

S4 Fig The *rpS12*^*D97*^ mutation affects life span. A-F) Survival curves of 3 replicates comparing *rpS12*^*G97D*^ flies with *w*^*11-18*^ (wild type) controls. 120 flies per sex per genotype per replicate. For A,C,E,F, P<0.0001; For B, p = 0.0012; For D, p = 0.0284 by Log-rank (Mantel-Cox) test. For raw data see the S6 Table.
(TIF)
