# Supplementary material for: Drosophila RpS12 controls translation, growth, and cell competition through Xrp1
Source: PLoS Genet. 2019 Dec 16;15(12):e1008513. doi: 10.1371/journal.pgen.1008513 (PMC6936874; doi:10.1371/journal.pgen.1008513)
Supplement: S1 Table — Fold changes (determined by DESeq2) in mRNA levels between wing discs from wild type and from indicated genotypes. Significant differences (Padj<0.05) indicated in bold. Genes shown here include all those corresponding to the enriched GO terms mature ribosome assembly (R), sulfur compound metabolic process (S), glutathione metabolic process (G), telomere maintenance (T), and DNA repair (D). These data correspond to the heatmap in Fig 1C. (PDF) [file pgen.1008513.s001.pdf]

gene	GO term	*rpS12^D97^*	*RpS17+/-*	*RpS3+/-*	*RpS3+/- rpS12^D97^*	*RpS3+/- Xrp1+/-*	*Xrp1+/-*
CG4858	S	1.03	**1.69**	**1.72**	1.13	1.18	1.07
CG8303	S	1.33	**1.82**	**2.30**	1.23	1.20	1.06
CG12264	S	0.91	**2.17**	**2.24**	0.94	0.98	0.86
CG17904	S	0.96	**1.62**	**1.64**	1.00	0.97	0.92
Ciapin	S	1.00	**1.70**	**1.77**	0.97	1.04	0.94
Iscu	S	**0.80**	**1.81**	**2.17**	**0.78**	0.93	0.91
Qtzl	S	0.95	**3.13**	**3.17**	1.06	1.10	0.95
Gclc	S,G	0.98	**2.07**	**2.05**	0.98	1.12	0.95
GstE1	S,G	**2.30**	**5.12**	**5.55**	**1.87**	**1.58**	1.07
GstE3	S,G	1.07	**4.48**	**2.61**	0.84	0.73	0.87
GstE5	S,G	**2.89**	**13.01**	**8.25**	**3.92**	**4.36**	1.96
GstE6	S,G	0.92	**36.44**	**42.08**	1.33	1.22	0.58
GstE7	S,G	2.00	**4.73**	**6.44**	0.84	0.50	0.73
GstE8	S,G	**0.46**	**2.16**	**2.15**	**0.36**	**0.46**	0.64
GstT1	S,G	0.94	**1.57**	**1.47**	0.89	0.90	1.01
ver	T	1.01	**4.31**	**3.68**	1.09	1.08	0.82
RpA-70	T,D	1.07	**1.60**	**1.71**	1.01	0.91	0.91
Irbp	T,D	1.00	**2.60**	**2.71**	0.87	0.81	0.87
Rad50	T,D	1.04	**1.52**	**1.57**	0.96	**0.82**	1.00
Ku80	T,D	**1.54**	**2.69**	**3.50**	**1.62**	**1.70**	1.25
Lig4	T,D	0.90	**1.62**	**1.63**	1.01	0.94	0.84
Mre11	T,D	**1.19**	**1.98**	**2.38**	**1.26**	1.04	0.96
Hus1-like	T,D	1.18	**2.06**	1.57	**1.59**	1.09	1.06
Mus205	D	0.94	**2.31**	**1.88**	**0.85**	**0.81**	0.90
Mus201	D	0.97	**1.43**	**1.49**	0.95	0.87	0.95
CG5181	D	**1.68**	**1.83**	**1.66**	**1.88**	**1.90**	**1.82**
CG6171	D	**1.23**	**1.68**	**1.71**	1.08	0.95	0.88
phr	D	**2.51**	**1.90**	**2.28**	**2.92**	**2.31**	1.20
Lig3	D	**0.72**	**2.36**	**2.28**	**0.69**	**0.57**	0.48
obe	D	0.96	**1.95**	**2.02**	1.01	0.91	0.91
agt	D	0.95	**1.64**	**1.65**	0.93	0.99	0.96
Xrp1	D	0.98	**2.42**	**2.41**	0.92	**0.84**	**0.71**
eIF6	R	0.94	**1.92**	**2.01**	0.99	1.04	0.94
CG8549	R	0.98	**1.57**	**1.80**	1.08	1.07	0.99
CG33158	R	0.95	**1.47**	**1.65**	0.96	0.96	0.92
rpS12		**1.55**	**0.62**	**0.62**	**1.32**	**0.56**	1.06
E(spl)m3		1.14	**0.79**	**0.64**	0.93	0.87	0.93
Socs36E		0.99	**1.47**	**1.28**	1.00	0.98	1.14
Upd3		0.79	**3.01**	2.23	0.55	0.69	0.92
