# Supplementary material for: Drosophila RpS12 controls translation, growth, and cell competition through Xrp1
Source: PLoS Genet. 2019 Dec 16;15(12):e1008513. doi: 10.1371/journal.pgen.1008513 (PMC6936874; doi:10.1371/journal.pgen.1008513)
Supplement: S2 Table — Fold changes (determined by DESeq2) in mRNA levels between wing discs from wild type and from indicated genotypes. Significant differences (Padj<0.05) indicated in bold. (PDF) [file pgen.1008513.s002.pdf]

gene	Pathway	*rpS12*^{D97}	*RpS17*$^{+/-}$	*RpS3*$^{+/-}$
brk	Dpp	1.04	1.01	0.98
tkv	Dpp	1.00	1.08	0.96
nkd	Wg	0.97	1.13	0.97
Fz3	Wg	1.07	0.98	0.96
Ptc	Hh	1.00	0.98	0.96
aos	Ras	0.97	1.11	1.04
E(spl)mβ	N	1.17	0.93	0.82
E(spl)mγ	N	1.46	1.19	1.32
E(spl)mδ	N	1.12	1.13	0.89
E(spl)m3	N	1.14	**0.79**	**0.64**
E(spl)m5	N	0.79	1.28	0.55
E(spl)m7	N	1.16	0.89	0.68
E(spl)m8	N	1.4	0.92	0.74
Socs36E	Jak/Stat	0.99	**1.47**	**1.28**
chinmo	Jak/Stat	1.59	0.91	1.29
puc	Jnk	1.01	1.19	1.08
Mmp1	Jnk	1.12	**1.64**	**1.32**
ex	SWH	1.10	1.14	1.06
Diap1	SWH	1.05	**1.22**	1.02
