# Supplementary material for: Drosophila RpS12 controls translation, growth, and cell competition through Xrp1
Source: PLoS Genet. 2019 Dec 16;15(12):e1008513. doi: 10.1371/journal.pgen.1008513 (PMC6936874; doi:10.1371/journal.pgen.1008513)
Supplement: S3 Table — (PDF) [file pgen.1008513.s003.pdf]

Transcription factor motifs enriched in introns and upstream region (5kb) of Rp-regulated genes (NES>3)

TF	NES	# Targets	# Motifs/Tracks
Xrp1	5.2941	55	10
Blimp-1	5.21899	45	18
Hsf	5.12023	63	13
lola	4.63761	47	3
CG9727	4.02362	19	6
grh	3.75515	57	4
Dip3	3.73948	23	3
slbo	3.71839	24	2
crp	3.56945	37	5
maf-S	3.24919	15	3
pnr	3.23289	44	8
Optix	3.08171	29	2
Hr38	3.05103	40	3
schlank	3.04975	11	1

Transcription factor motifs enriched in 5' UTR, first intron, and upstream region (5kb) of Rp-regulated genes (NES>3)

TF	NES	# Targets	# Motifs/Tracks
Blimp-1	5.53166	33	5
lola	5.44284	48	4
crp	5.3291	35	6
**Xrp1**	**4.66517**	**53**	**15**
CG6272	4.39951	41	6
slbo	4.3959	46	3
grh	4.19133	61	6
E2f	4.14712	57	2
ewg	4.04062	33	3
sqz	3.82802	19	4
CG9727	3.75688	15	2
Pur-alpha	3.65641	21	3
Side	3.43576	36	4
cnc	3.32524	35	4
Atf3	3.26375	30	3
prd	3.1042	17	2
Tbp	3.09697	22	4
ERR	3.05356	32	6
CG17829	3.03347	17	1

Rp-regulated genes with Xrp1-binding motifs according to one or both analyses

Ady43A	amd	baz	beat-IIb	cac	Cda5
CG1090	CG12099	CG12384	CG13258	CG13579	CG13731
CG14291	CG14304	CG15080	CG15784	CG1673	CG18547
CG2781	CG3036	CG31140	CG3168	CG32512	CG32694
CG33158	CG33494	CG34382	CG3726	CG3902	CG4858
