# Supplementary figures and images for: Drosophila RpS12 controls translation, growth, and cell competition through Xrp1
Source: PLoS Genet. 2019 Dec 16;15(12):e1008513. doi: 10.1371/journal.pgen.1008513 (PMC6936874; doi:10.1371/journal.pgen.1008513)

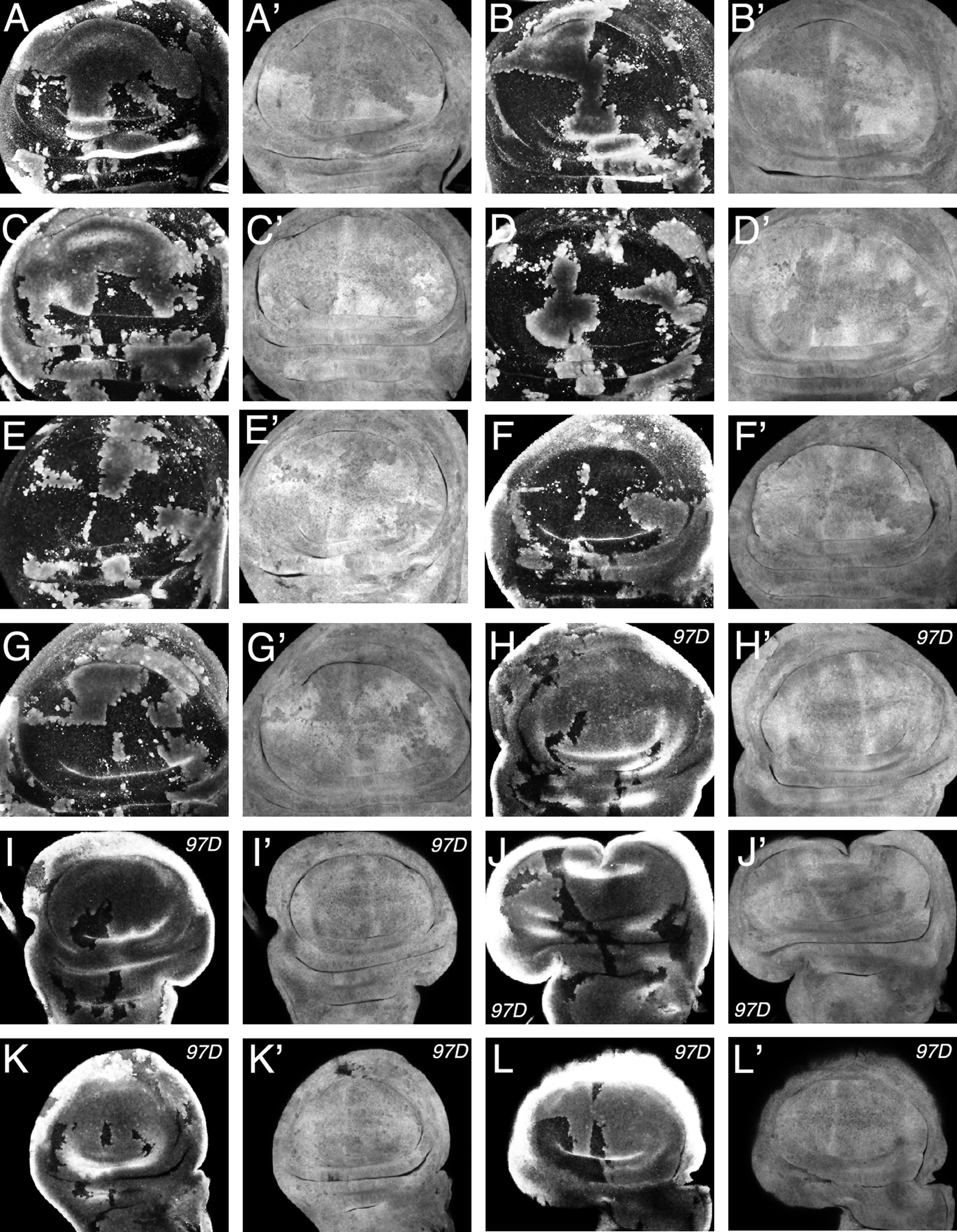

Supplement: S1 Fig — Comparing translation rates measured by OPP incorporation between different regions of wing discs is complicated to some extent by the dynamic patterns of translation that occur the in wild type [16], perhaps reflecting the dynamic and patchy activity of TORC1 that is revealed by RpS6 phosphorylation patterns[58]. Changes due to mutations in Rp genes are superimposed upon this variable background and are best assessed by directly observing how translation changes cell-autonomously along sharp clone boundaries. In support of the conclusion that RpS17 mutations reduce translation in an RpS12-dependent manner (see Fig 2E–2H), we present additional examples of cell autonomous differences in translation rate between RpS17+/- cells, labeled with GFP, and unlabeled RpS17+/+ clones (panels A-G). Translation, shown by OPP incorporation in panels A’-G’, is consistently lower in RpS17+/- regions. In contrast to these rpS12+/+examples, clones of RpS17+/+ rpS12D97/D97cells in RpS17+/- rpS12D97/D97wing discs (panels H-L) show no difference in translation rate (panels H’-L’). (TIF) [file pgen.1008513.s009.tif]

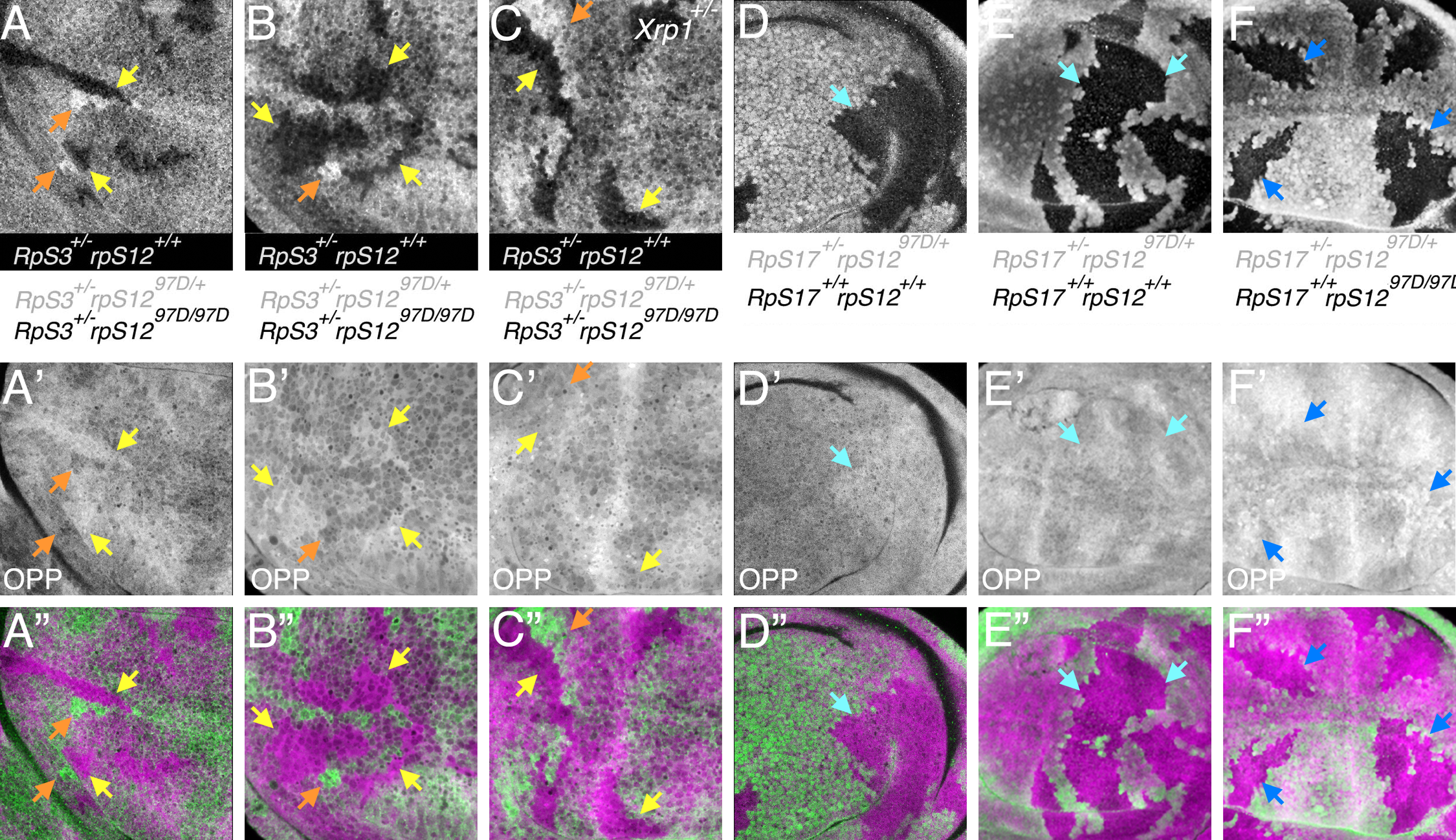

Supplement: S2 Fig — Panels A-F show wing discs containing clones of indicated genotypes. Corresponding translation rate (OPP incorporation) is shown in panels A’-F”, and the overlay of translation and genotype in panels A”-F”. A,B,C indicated that rpS12 had cell-autonomous, Xrp1-dependent effects on translation in RpS3+/- cells. (A-A” and B-B”) RpS3+/- rpS12+/+ clones often show lower translation than the RpS3+/- rpS12D97/+ background (eg orange arrows). Translation was often higher in RpS3+/- rpS12D97/D97 clones (eg yellow arrows). (C-C”). In the Xrp1+/- background, translation rates were unaffected by rpS12 genotype. D,E,F show RpS17+/- genotypes. Because rpS12 and RpS17 both map to chromosome 3L, FLP-mediated recombination cannot generate RpS17+/- rpS12D97/D97 and RpS17+/- rpS12+/+ clones in the same disc. These figures show that, unlike RpS17+/- rpS12+/+ cells (see main text Fig 2E and 2F), translation in the RpS17+/- rpS12D97/+ genotype was only sometimes distinguishable from that of RpS17+/+ cells. (D-D”). In some cases, RpS17+/+ rpS12+/+ clones showed translation rates higher than the RpS17+/- rpS12D97/+ background (eg cyan arrows). (E-E”). In most cases, RpS17+/+ rpS12+/+ clones typically showed translation rates similar to the RpS17+/- rpS12D97/+ background (eg cyan arrows). (F-F”) Little or no translation difference was seen between RpS17+/+ rpS12D97/D97 clones and the RpS17+/- rpS12D97/+ background (eg blue arrows). Genotypes used. A,B) y w hsF; rpS1297D FRT80B M(3)95A /rpS1297D FRT80B P{arm-LacZ}. C) y w hsF; rpS1297D FRT80B M(3)95A/rpS1297D FRT80B P{arm-LacZ} Xrp1m2-73. D,E) y w hsF; RpS174 rpS1297D P{ubi-GFP} FRT80B/FRT80B. F) y w hsF; RpS174 P{ubi-GFP} FRT80B/rpS1297D FRT80B. (TIF) [file pgen.1008513.s010.tif]

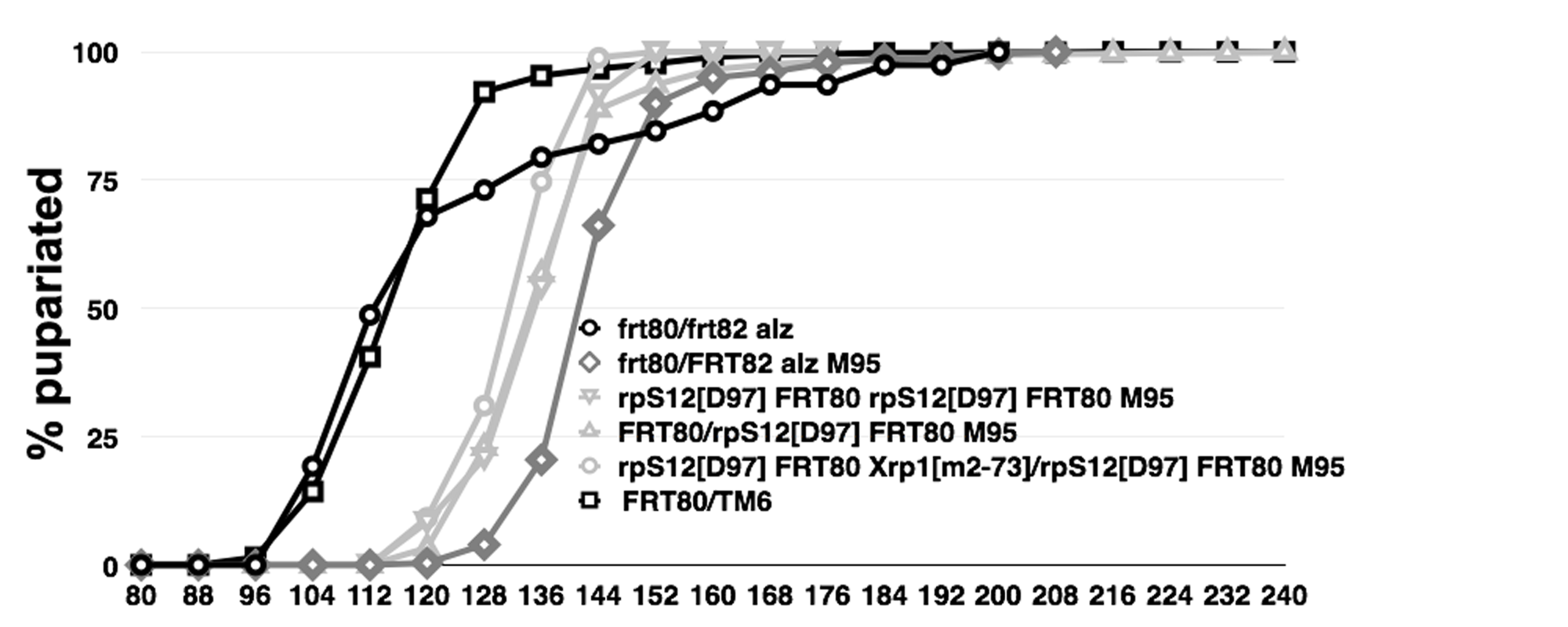

Supplement: S3 Fig — The timing of pupariation was measured in the same experiment shown in Fig 3K and 3L. Like the effects on adult emergence, rpS12D97/D97 and rpS12D97/+ genotypes suppressed the delay to pupariation on RpS3+/- larvae, to a similar extent to RpS3+/- rpS12D97/D97 Xrp1+/- larvae. The detailed genotypes used and numerical data corresponding to these graphs is tabulated in S5 Table. (TIF) [file pgen.1008513.s011.tif]

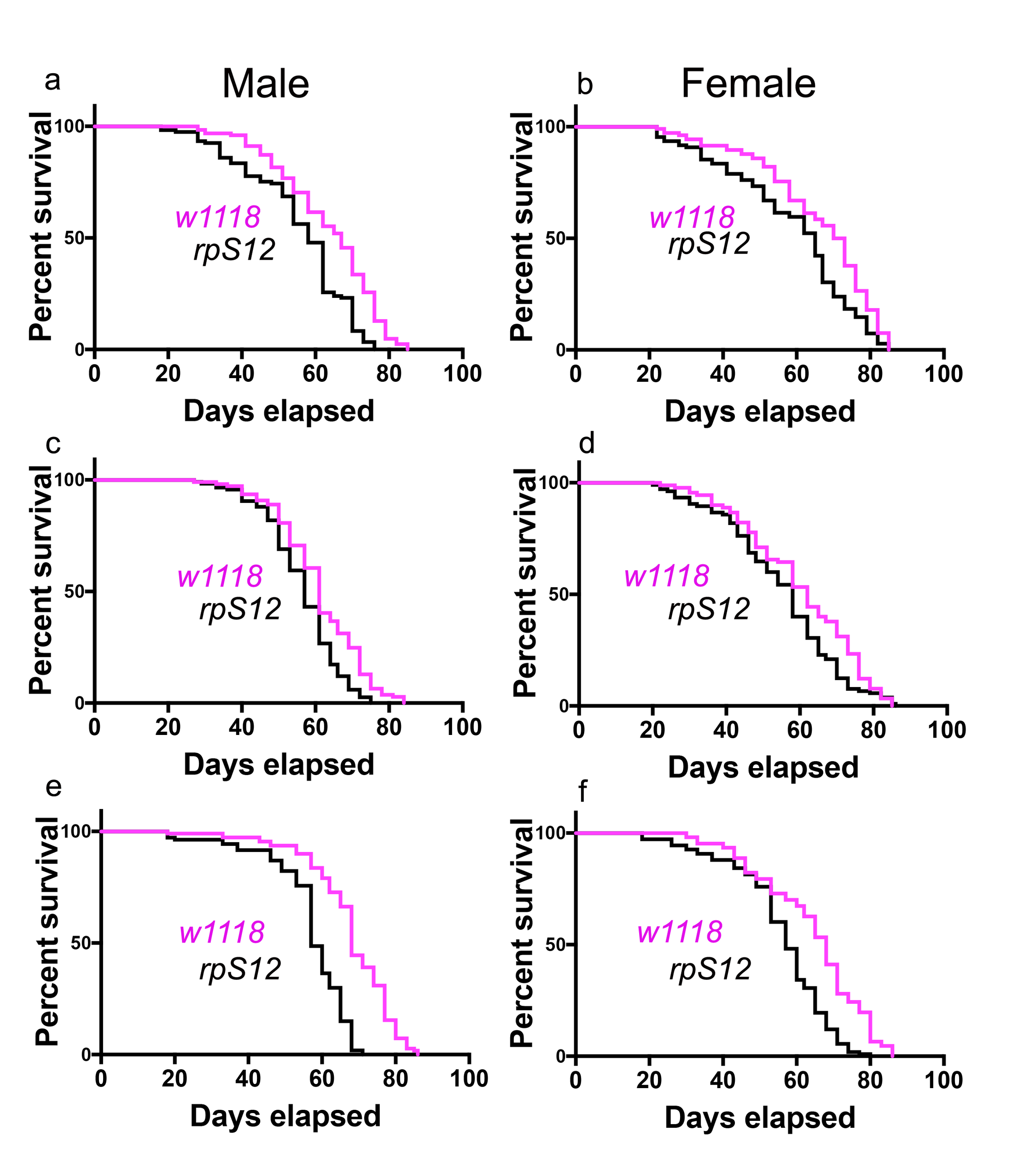

Supplement: S4 Fig — A-F) Survival curves of 3 replicates comparing rpS12G97D flies with w11-18 (wild type) controls. 120 flies per sex per genotype per replicate. For A,C,E,F, P<0.0001; For B, p = 0.0012; For D, p = 0.0284 by Log-rank (Mantel-Cox) test. For raw data see the S6 Table. (TIF) [file pgen.1008513.s012.tif]
